# Uptake and Effectiveness of Intermittent Preventive Treatment with Sulfadoxine-Pyrimethamine during Pregnancy in Africa: A Scoping Review

**DOI:** 10.3390/diseases12090203

**Published:** 2024-09-04

**Authors:** Gifty Osei Berchie, Patience Fakornam Doe, Theodora Dedo Azu, Joyce Agyeiwaa, Gifty Owusu, Christian Makafui Boso, Naomi Kyeremaa Yeboa, Dorcas Frempomaa Agyare, Irene Korkoi Aboh, Bernard Nabe, Godson Obeng Ofori, Benjamin Anumel, Justice Enock Kagbo, Amidu Alhassan, Frank Odonkor Offei, Rita Opoku-Danso, Susanna Aba Abraham, Mustapha Amoadu, John Elvis Hagan

**Affiliations:** 1 Department of Maternal and Child Health, School of Nursing and Midwifery, College of Health and Allied Sciences, University of Cape Coast, PMB, Cape Coast CC 3321, Ghana; gberchie@ucc.edu.gh (G.O.B.); theodora.azu@ucc.edu.gh (T.D.A.); joyce.agyeiwaa@ucc.edu.gh (J.A.); gifty.owusu@ucc.edu.gh (G.O.); naomi.yeboa@ucc.edu.gh (N.K.Y.); iaboh@ucc.edu.gh (I.K.A.); 2 Department of Public Health, School of Nursing and Midwifery, College of Health and Allied Sciences, University of Cape Coast, PMB, Cape Coast CC 3321, Ghana; patience.doe@ucc.edu.gh (P.F.D.); sabraham@ucc.edu.gh (S.A.A.); 3 Department of Adult Health, School of Nursing and Midwifery, College of Health and Allied Sciences, University of Cape Coast, PMB, Cape Coast CC 3321, Ghana; christian.boso@ucc.edu.gh (C.M.B.); dorcas.agyare@ucc.edu.gh (D.F.A.); bnabe@stu.ucc.edu.gh (B.N.); gofori002@stu.ucc.edu.gh (G.O.O.); justicekagbo@gmail.com (J.E.K.); amidualhassan24@gmail.com (A.A.); fodonkor36@gmail.com (F.O.O.); rita.opoku-danso@ucc.edu.gh (R.O.-D.); 4 Center for Health Research and Policy Innovations, Legon, Accra P.O. Box LG 949, Ghana; bs.anumel@gmail.com; 5 Biomedical and Clinical Research Centre, College of Health and Allied Sciences, University of Cape Coast, PMB, Cape Coast CC 3321, Ghana; mustapha.amoadu@ucc.edu.gh; 6 Department of Health, Physical Education and Recreation, University of Cape Coast, PMB, Cape Coast CC 3321, Ghana; 7 Neurocognition and Action-Biomechanics-Research Group, Faculty of Psychology and Sports Science, Bielefeld University, Postfach 10 01 31, 33501 Bielefeld, Germany

**Keywords:** IPTp-SP, uptake, pregnancy, effectiveness, Africa, scoping review

## Abstract

Malaria poses a significant threat to pregnant women in sub-Saharan Africa, necessitating effective interventions like the intermittent preventive treatment of malaria in pregnancy with sulfadoxine-pyrimethamine (IPTp-SP). However, challenges persist in the uptake and effectiveness of this intervention. This scoping review aims to explore IPTp-SP uptake in African countries, identify influencing factors, and assess its effectiveness in preventing malaria and adverse outcomes in pregnancy. This scoping review follows Arksey and O’Malley’s framework, employing the PRISMA-ScR guidelines for reporting. Searches were conducted in PubMed, Embase, Scopus, JSTOR, Web of Science, Google Scholar, and ProQuest, focusing on studies post-2000 published in the English language. The search produced 15,153 records, of which 104 full-text records were eligible and 101 papers were included in this review. The findings suggest varying IPTp-SP uptake rates, spanning from 5.3% to 98.9%, with their effectiveness supported by longitudinal studies, randomised controlled-trials (RCTs), cross-sectional surveys, and mixed-method studies. IPTp-SP demonstrates efficacy in reducing malaria during pregnancy, placental parasitaemia, and anaemia episodes, alongside improved birth outcomes. Common adverse effects of IPTp-SP include prematurity and low birth weight. Facilitators of IPTp-SP uptake include education and ANC attendance, while commonly reported barriers included inadequate knowledge and healthcare system challenges. The findings also suggest adverse effects such as prematurity, low birth weight, and maternal and perinatal mortality associated with IPTp-SP uptake. It is vital to strengthen antenatal care services by integrating comprehensive counselling on IPTp-SP and address healthcare system challenges. Community engagement, women’s empowerment, and context-specific interventions are necessary for promoting IPTp-SP uptake and improving maternal and neonatal health outcomes in Africa.

## 1. Introduction

Malaria, a formidable global health challenge, disproportionately affects economically disadvantaged regions, especially in Africa. In 2021 alone, Africa accounted for 95% of the 247 million reported cases and nearly 96% of the associated deaths [1,2]. Vulnerable populations, notably pregnant women and young children in endemic regions, face heightened risks, evidenced by an annual incidence of approximately 25 million cases among pregnant women in Africa, significantly amplifying the risk of maternal mortality [3,4,5,6]. This pressing issue requires urgent attention and innovative, equitable solutions to alleviate the impact on the most vulnerable members of our global community.

Pregnancy exacerbates women’s susceptibility to severe malaria by compromising their immune system [7,8]. This heightened vulnerability, which can lead to severe anaemia and gestational hypertension, results in a high mortality rate approaching 50% among pregnant women in malaria-endemic regions [4,6,9,10]. Moreover, the impact of malaria during pregnancy extends to significant risks for the developing foetus, elevating the likelihood of preterm birth and a low birth weight due to intrauterine growth restriction [7], with an estimated 900,000 low-birth-weight deliveries globally attributed to malaria, of which about 100,000 resulted in newborn mortality [11]. Surviving infants with a low birth weight face an increased risk of impaired intellectual and social development in later years, emphasizing the urgent need for targeted interventions in malaria-endemic regions [12,13,14]. The intricate interplay of these factors underscores the necessity for comprehensive strategies to safeguard maternal and neonatal well-being.

The World Health Organization (WHO) implemented a range of measures in 2000 to assess the effects of malaria during pregnancy. One of the main interventions was the use of intermittent preventive treatment in pregnancy (IPTp) with sulfadoxine-pyrimethamine (SP), which is highly recommended [15,16,17,18]. IPTp-SP is a malaria-prevention strategy where pregnant women take doses of SP at scheduled intervals to reduce the risk of malaria infection and its associated complications [17,18]. Despite these efforts to protect pregnant women in Africa against malaria, recent analyses suggest that Africa is falling short of its malaria morbidity and mortality targets, achieving only 45% and 47% of the indicators, respectively [17]. This shortfall raises concerns about the United Nations’ Sustainable Development Goal 3, which aims to reduce the maternal mortality ratio to less than 70 per 100,000 live births by 2030.

Recognizing the potential of IPTp-SP to enhance maternal and neonatal health, as emphasized by the WHO in 2012, it is essential to acknowledge persistent challenges in its uptake, accessibility, and effectiveness, as highlighted in a recent study by Nana et al. [19]. Despite over two decades of research on IPTp-SP in Africa, comprehensive reviews that aim to synthesize and map these studies remain scarce. The existing reviews have primarily focused on different aspects of IPTp. For instance, van Eijk et al. [20] examined the resistance of IPTp-SP, Egbujor et al. [21] discussed interventions aimed at improving its uptake, and Kayentao et al. [22] evaluated the effectiveness of two versus three or more doses of SP on the risk of low birth weight in Africa. However, these reviews did not address the prevalence of and factors influencing the uptake of IPTp-SP in Africa. Moreover, these reviews included few studies, probably due to the focus on experimental designs, interventional studies, and, also, stringent eligibility. Additionally, Hill et al. [23] explored barriers to the uptake of both IPTp-SP and insecticide-treated nets (ITNs) without a sub-group analysis specifically focusing on barriers to IPTp uptake. Another review by Sylla et al. [24] attempted to address this issue but did exclusively consider health system barriers affecting the uptake of IPTp in Africa. There is a clear need for a review methodology that allows a comprehensive mapping of the evidence on the prevalence of, facilitators of, barriers to, and effectiveness of IPTp-SP, the common type of IPT treatment for the prevention of malaria in pregnancy in sub-Saharan Africa (SSA). This approach will provide a more holistic understanding of the factors influencing the uptake of IPTp-SP and offer insights into how to improve its implementation and effectiveness.

Therefore, a scoping review is crucial to thoroughly investigate the uptake of IPTp-SP in Africa, discern the factors that influence access to this intervention, and consolidate the existing body of evidence regarding its effectiveness in preventing malaria and mitigating associated adverse outcomes. The evidence from this review will emerge as a valuable resource for healthcare service providers, policymakers, and stakeholders, delivering valuable insights into utilization levels, barriers to access, and outcomes associated with IPTp-SP in the African context. Furthermore, evidence from this review will help develop context-specific interventions that have a high potential for improving the accessibility and uptake of IPTp-SP in Africa. This review will inform future studies and systematic reviews by highlighting the gap and kind of evidence that exist on this subject. Thus, the insights into the factors influencing access to IPTp-SP, its effectiveness, and barriers to its utilization in Africa may help in conducting a robust systematic review and meta-analysis.

The socio-ecological framework [25] was used as a framework in organising the factors (facilitators and barriers) that influence the uptake of IPTp-SP. The socio-ecological framework, proposed by Bronfenbrenner in 1994, offers a comprehensive approach to understanding human behaviour within the context of various environmental systems. This model identifies multiple levels of influence, including the individual, interpersonal, community, organizational, and societal levels, and emphasizes the dynamic interactions between these levels. In the context of IPTp-SP uptake, the socio-ecological model allows for the exploration of factors at each of these levels that either facilitate or impede the adoption of the intervention. The strength of the socio-ecological model lies in its holistic approach to understanding health behaviours by considering the complex interplay between individual, interpersonal, and environmental factors. By examining factors across multiple levels of influence, the model allows for a more comprehensive understanding of the determinants of IPTp-SP uptake. This facilitates the development of multi-level interventions that address barriers at various levels, leading to more effective strategies for improving IPTp-SP coverage and ultimately reducing the burden of malaria in pregnancy.

## 2. Methods

### 2.1. Research Design

This review followed the framework proposed by Arksey and O’Malley, [26], which consists of six consecutive stages: (1) formulating the research question, (2) identifying relevant studies, (3) selecting studies, (4) organising data, (5) compiling, summarising, and reporting results, and (6) seeking consultation. The reporting of this review followed the PRISMA-ScR checklist by Tricco et al. [27]. Utilisation of PRISMA-ScR ensured a structured and comprehensive approach to reporting that aligns with best practices in scoping review methodologies. This review utilised primary studies that had ethical clearance and followed appropriate ethical standards in conducting their studies. Furthermore, this scoping review protocol has been registered in the Open Science Framework with the number doi:10.17605/OSF.IO/UNC7R.

### 2.2. Research Questions

The following research questions guided this review:

1. What is the level of IPTp-SP uptake during pregnancy in Africa?
2. What are the facilitators and barriers to IPTp-SP access among pregnant women in Africa?
3. What is the effectiveness of IPTp-SP among pregnant women in Africa?
4. What are the adverse effects of IPTp-SP on maternal and neonatal outcomes in Africa?

### 2.3. Search Strategy

A comprehensive search was performed in five databases, namely PubMed, Embase, Scopus, JSTOR, and Web of Science. Researchers [28] have stated that these specific databases contain a diverse array of the relevant literature on health. The authors created a search strategy by employing a combination of regulated terminologies like medical subject headings (MeSH) and specific keywords for conducting searches in the designated databases. A preliminary search was conducted in PubMed (See Table 1), and the MeSH terms were further refined for searching in the remaining databases. Moreover, an extra hand search was conducted on Google, ProQuest, OSF Registries, the WHO repository, and Google Scholar to collect additional relevant literature. Lastly, the reference lists of eligible studies were manually checked for additional eligible records. The last search was conducted on 13 May 2024.

This table presents our comprehensive search strategy to identify studies on the uptake and effectiveness of intermittent preventive treatment in pregnancy with sulfadoxine-pyrimethamine (IPTp-SP) in Africa. The first part of the table shows the planned search strategy which contains the lists of search terms for various concepts, including IPTp-SP and its uptake and effectiveness, pregnancy, and a geographical focus on Africa. The second part of the table details the actual search strategies used in specific databases like PubMed, Embase, Scopus, Web of Science, and JSTOR. Each entry describes the precise search strings and keywords employed to identify relevant studies on the uptake and effectiveness of IPTp-SP, ensuring a thorough and systematic review of the literature. It is worth noting that not all planned search terms were used together in every database search. Instead, the search was adjusted to fit each database’s system, which sometimes required a more focused approach to get the best results. For example, during a manual search for specific papers, we used the names of individual countries instead of “Africa” to find relevant studies that might have been missed using a broader term.

### 2.4. Inclusion Criteria

This review included all studies conducted on IPTp-SP in African individuals. Studies published in English were utilized to ensure easier comprehension by the authors. The proposed review included studies published in 2000 and later because the WHO introduced the use of IPTp-SP as a prophylactic treatment for malaria in 2000 [17,29]. The authors incorporated both peer-reviewed and grey studies from the literature to guarantee comprehensive retrieval of all pertinent papers applicable to this review.

### 2.5. Exclusion Criteria

The authors excluded studies that specifically examined IPTp-SP with people from continents other than Africa. Preprints, abstracts, letters to editors, commentaries, and reviews were excluded from this review.

### 2.6. Study Selection

The authors uploaded the identified articles into the Mendeley software (Version 1.19.3) and eliminated duplicates. Titles and abstracts were screened by 25 trained postgraduate students. Three of the authors (SAA, TDA, MA) oversaw and guided this process. The screening of titles and abstracts was done to get only full-text eligible records. Reference lists of full-text eligible studies were checked for additional papers. Subsequently, the authors divided themselves into two groups, namely (GOB, TDA, ROD, JA, JK, GO) and (PFD, CO, DFA, FN, NKY, CMB), and independently evaluated the full-text studies based on the specified criteria for inclusion. The groups addressed and resolved their disagreements in weekly sessions. The screening procedure was overseen by MA and SAA. The use of two groups for the evaluation of full-text studies and the resolution of disagreements in weekly sessions further strengthened the reliability of the review. The PRISMA flow diagram was utilized to report the search results from each database, screening process, and screening results.

### 2.7. Data Extraction

The authors created a framework for extracting relevant information. The data that were extracted included information such as the authors, publication year, study location, study objective, study design, demographic, sample size, uptake level, barriers and facilitators, effectiveness, and adverse effects. This form was tested to collect data from four studies. Following that, two sets of authors (GOB, TDA, ROD, JA, JK, GO) and (PFD, CO, DFA, FN, NKY) independently extracted the data from the included studies. Other authors (AAD, MA, and SAA) reviewed the extraction. To guarantee the quality and accuracy of the retrieved data, the authors and reviewers scheduled weekly meetings to address any inconsistencies and other challenges.

### 2.8. Collating, Summarising and Reporting the Results

The characteristics of included studies were subjected to a thorough analysis using Microsoft Excel, resulting in the generation of tables, charts, and maps to visually depict important information. Thematic analysis and synthesis of findings from included studies were done. The authors thoroughly engaged with the retrieved data through extensive reading. This enabled them to formulate the first codes that effectively capture the core information. The initial codes were categorized into overarching themes and subthemes, each carefully defined and labelled, creating an organized structure that directed the subsequent reporting process. Narrative reporting of the findings was done.

### 2.9. Consultation

As outlined in the framework of Arksey and O’Malley [26], consultation occurred at every stage of the review procedure. A certified librarian was consulted during the search and screening of relevant studies. The proposed evaluation sought inputs from the directors of the Malaria Control Programme regarding any pertinent material and information. Also, mothers who have ever taken the IPTp-SP were consulted for their input during the conceptualisation of the review and discussion of the findings. Consultations were undertaken to ensure a comprehensive review, gather diverse perspectives, and incorporate valuable insights from stakeholders to enhance the quality and relevance of the evaluation process. These consultations aimed to enrich the review by accessing a wide range of source from the literature, expert opinions, and lived experiences, thereby strengthening the validity and applicability of the findings and recommendations.

## 3. Results

### 3.1. Search Results

**Figure 1:**
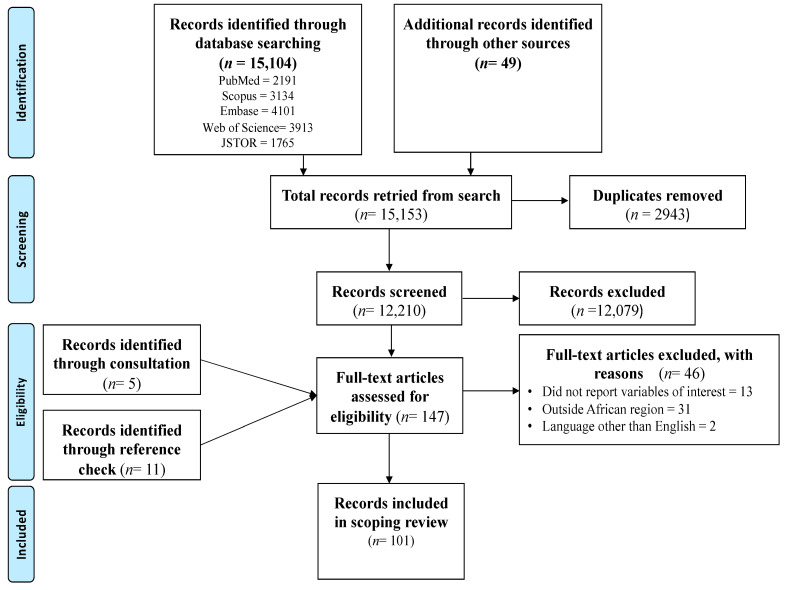
PRISMA flow chart of the search results and screening process.

The search conducted in the five main databases produced 15,104 records. An additional 49 records were retrieved from searches conducted in other databases. The use of the Mendeley software allowed the deletion of 2943 duplicate records. The screening of the remaining 12,210 titles and abstracts led to the exclusion of 12,079 records. These records were not eligible and were not related to the variables of interest, were outside the geographical area and population, or did not focus on pregnant women. After screening of the titles and abstracts, 131 full texts were eligible for further screening. Checking the reference list resulted in an additional 11 full-text eligible records while consultations with a digital librarian resulted in the retrieval of 5 additional records. Hence, 147 full-text eligible records were further screened for inclusion. Finally, 46 records were excluded with reasons and 101 records were included in this scoping review. See Figure 1 for the PRISMA flow chart.

### 3.2. Characteristics of Included Studies

**Figure 2:**
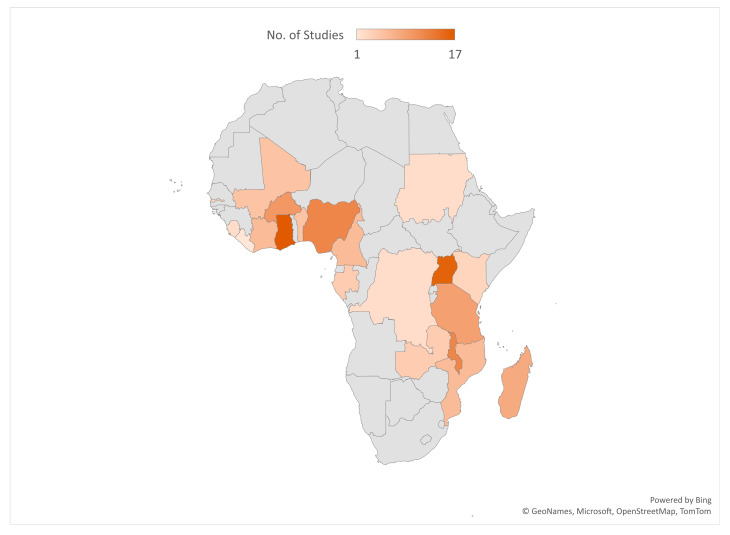
Countries where included studies were conducted.

**Figure 3:**
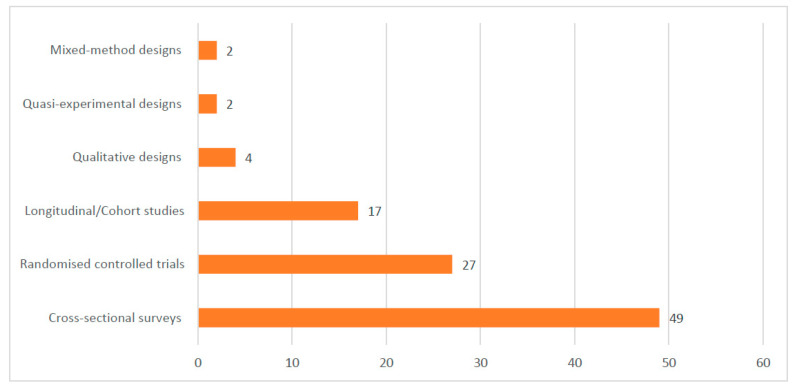
Designs used in the included studies.

**Figure 4:**
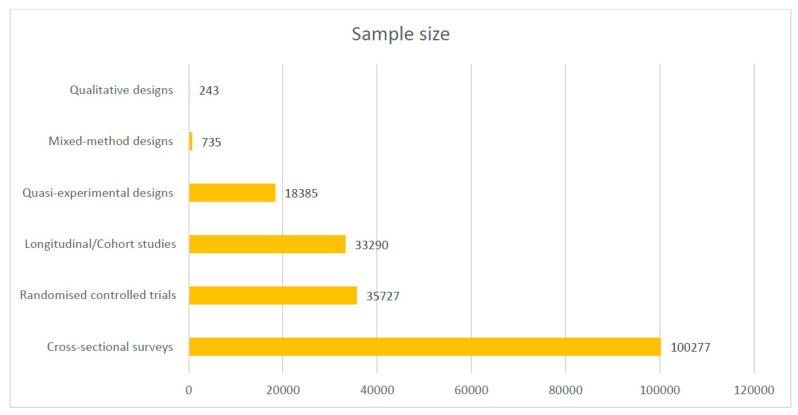
Sample size of included studies according to designs.

Most of the included studies were conducted in Ghana (17), Uganda (16), Malawi (12), Nigeria (12), and Burkina Faso (10). See the details of where the studies were conducted in Figure 2. Out of the 101 included studies, 49 were cross-sectional surveys. See Figure 3 for details on the study types. A total of 188,657 samples were used in the included studies. Details on the sample size according to the different study designs are presented in Figure 4.

### 3.3. Rate of Uptake of IPTp-SP Based on Reported Doses

Thirty-seven (37) studies reported on the rate of IPTp-SP uptake based on the number of doses. For at least one dose of IPTp-SP, the uptake rates ranged from 5.3% [30] to 98.9% [31]. For at least two doses of IPTp-SP, the uptake rates ranged from 15.0% [32] to 95.5% [33]. At least three doses of IPTp-SP had reported uptake rates ranging from 6.0% [34] to 93.2% [35]. Additionally, for at least four doses or more, the uptake rates ranged from 7.5% [36] to 42.3% [30]. Finally, the uptake rate for five doses or more was reported as 17.1% by Vandy et al. [31]. The uptake rates were lower for studies that reported on at least three doses or more compared to studies that focused one or two doses. Thus, the IPTp-SP uptake rate decreases with the number of doses. Studies that sampled participants in healthcare settings like ANC facilities [37,38,39] and delivery units [40] generally reported higher IPTp-SP uptakes compared to studies that relied on populations from community settings or the general population. See details in Table 2.

### 3.4. Factors Affecting the Uptake of IPTp-SP

The factors affecting the influence of IPTp-SP were put into two main categories, facilitators and barriers. Each category was put into the socio-ecological model.

#### 3.4.1. Facilitators of IPTp-SP Uptake

Forty-two (42) studies reported on factors that facilitate the uptake of IPTp-SP in SSA [31,34,35,36,37,45,46,48,50,51,52,53,54,55,56,57,59,61,62,63,64,65,68,69,70,71,72,73,74,75,76,77,78,79,80,81,82,83,84,85,86]. These factors were organised according to the socio-ecological framework.

##### Individual Factors

For the facilitation of IPTp-SP uptake, several factors have been identified as influential. Regarding individuals’ educational levels, any level of education can play a role [59,71,72]. However, Mushi et al. [61] specifies that primary education may enhance IPTp-SP uptake. Similarly, while some studies highlight the positive impact of secondary or higher education on IPTp-SP utilization [34,35,73,74], others indicate that individuals with no education have higher chances of utilizing IPTp-SP [53]. This demonstrates that evidence on the influence of education has been inconsistent across studies. Although age has been reported as a significant predictor of IPTp-SP, this has been inconsistent across studies. For instance, the uptake is higher among women aged 15–29 years [49,54,75,76]. Other studies reported a higher uptake among women aged 30–39 years [72,75,77]. Additionally, being married or living with a partner is stressed as significant in facilitating IPTp-SP usage [75].

Comprehensive knowledge about IPTp-SP, as highlighted by [31,48,49], is associated with a better uptake. Understanding prophylaxis for malaria, as demonstrated in a study by [78], helps pregnant women make informed choices. Sangho et al. [59] emphasizes the importance of knowing the recommended dose of IPTp-SP, which improves its effectiveness. Awareness of malaria risks during pregnancy, as underscored by Azizi et al. [51], is crucial for prevention. The promotion of awareness about malaria [75] and IPTp-SP [55] encourages uptake. Furthermore, exposure to media messages, as demonstrated by [36,37,71], Barrow et al. [53], and [56], spreads information and encourages IPTp-SP use.

Knowledge about IPTp-SP, including its prophylaxis and recommended dose, contributes to its uptake [31,48,49,59,78]. Studies also suggest that awareness of malaria in pregnancy and its dangers [51], as well as awareness about malaria-prevention programs and IPTp-SP [55,75], positively influence IPTp-SP uptake. ANC attendance in the first trimester [61] or the second trimester [60,84] improves the uptake of IPTp-SP. Thus, the early initiation of ANC [59] in a government health facility [61] is crucial for IPTp-SP uptake. Having three [59] or four or more [51,52,56,61,63] ANC visits is positively associated with IPTp-SP uptake. Other facilitators include having three or more children [49], being a woman with advanced pregnancy (24–40 weeks) [77], and being a woman who tested positive for malaria [77]. Anaemic pregnant women [77] and those who use insecticide-treated nets (ITNs) [68] also show an improved IPTp-SP uptake.

##### Interpersonal Factors

Higher levels of education among husbands [74] and being part of a richer household [34,65,71] are significant predictors of IPTp-SP uptake. Included studies also indicate that educating men about the dangers of malaria in pregnancy [70] and providing education and counselling on malaria during antenatal care (ANC) attendance [79] improve the uptake of IPTp-SP.

##### Institutional Factors

The institutional facilitators include the proximity of health facilities [51], trust in the healthcare system [80], the continuous training of healthcare providers [70], high stock levels of IPT-SP [63,79], and the distribution of IPT-SP during ANC [69,74]. Additionally, utilizing the directly observed treatments (DOTS) strategy [81], the wider coverage of ANC [80], and increased ANC attendance [35,45,57] have been identified as predictors of IPTp-SP uptake. Exposure to media messages also plays a significant role in promoting awareness and the uptake of IPTp-SP [36,37,53,56,71].

##### Community/Societal Factors

The community/societal facilitators include factors such as rural dwelling [50], residence in urban areas [82], the encouragement of social networks [80], favourable social norms for IPTp-SP [83], and community sensitization efforts [84]. Additionally, the community delivery/distribution of IPTp-SP [79,87], the presence of health centres [59], and proximity to the nearest health centre [84] also play a role in facilitating IPTp-SP uptake. See details in Table 3.

#### 3.4.2. Barriers to IPTp-SP Uptake

Thrity-two (32) studies reported on barriers to the uptake of IPTp-SP in SSA [32,34,35,36,48,50,51,54,55,57,63,65,66,76,77,80,81,82,83,84,88,89,90,91,92,93,94,95]. These factors were organised according to the socio-ecological framework.

##### Individual

The barriers to IPTp-SP uptake encompass various individual factors which contribute to poor knowledge and awareness. Studies indicate that women with inadequate knowledge about IPTp-SP [94,95] are less likely to complete the recommended dose of IPTp-SP [36]. Factors such as fear of side effects [81], the perception of adverse effects on pregnant women [93], and refusal to take SP [80] also hinder uptake. Late initiation and irregular attendance of ANC appointments contribute to suboptimal IPTp-SP use [80,85,94], alongside women who receive the first dose in the third trimester [63] and multigravida women being less likely to complete the recommended dose [43]. A higher parity [35,65], HIV-positive status [81], history of child death [42], and lower educational attainment [42,50,55] also serve as barriers. Additionally, being single [42] and belonging to poor socioeconomic backgrounds [54] are associated with reduced IPTp-SP uptake.

##### Interpersonal

Interpersonal barriers to IPTp-SP uptake often revolve around household economic and social factors. Studies indicate that households facing economic challenges, such as the poorest households [79,92] and those with a lower socioeconomic status [65], may encounter difficulties in accessing IPTp-SP. Additionally, issues related to autonomy and decision-making power within households can impede IPTp-SP uptake, particularly among women who lack the freedom to make health-related choices [66].

##### Institutional Factors

The institutional barriers to the uptake of IPTp-SP are multifaceted and encompass various challenges within healthcare systems and provider practices. One significant obstacle is the lack of adequate training and supervision for healthcare workers, leading to inconsistencies in IPTp-SP administration [91]. Moreover, inadequate information being provided to women about IPTp-SP during antenatal care visits further hampers its uptake [48,80,85]. Another issue is the inadequacy of routine training for healthcare providers, which contributes to gaps in knowledge and practice regarding IPTp-SP [48]. Healthcare provider’s refusal to provide services [91] and high staff turnover rates also pose significant challenges [90]. Additionally, uncertainty among healthcare providers about the safety and efficacy of IPTp-SP can influence their recommendations to pregnant women, further impacting its uptake [91]. The inconsistent provision of antenatal care services [93] and perceptions that IPTp-SP has adverse effects on pregnant women also contribute to a low uptake [91]. Moreover, inadequate counselling or support for pregnant women during ANC visits exacerbates the problem [92]. Financial barriers, including ANC costs and health financing challenges, further limit access to IPTp-SP for vulnerable populations [77,80]. Frequent stockouts of SP and other essential supplies at healthcare facilities are additional barriers to IPTp-SP uptake, resulting in missed opportunities for administration [57,66,82,89,92].

Insufficient information provided to women about IPTp-SP during antenatal care visits is a significant issue [48,80,85]. Furthermore, misinformation about the standard doses of IPTp-SP persists, hindering effective administration [93]. A lack of comprehensive knowledge of IPTP policy among healthcare providers further complicates the situation [77], as does the inadequate supply of SP to the health sector, leading to stockouts and missed opportunities for administration [91]. Demand and supply bottlenecks exacerbate the problem, creating challenges in accessing IPTp-SP [77]. Moreover, inadequate supply for the private sector limits access to SP in certain settings [91]. Limited healthcare infrastructure, including long waiting times at ANC clinics, presents additional barriers to IPTp-SP uptake [89,92]. The poor implementation of directly observed therapy strategies further hinders effective administration [66]. Non-institutional births, inconsistent guidelines for IPTp, and inadequate guidelines for healthcare providers also contribute to low uptake rates [91]. The geographical challenge of having a working distance of more than 30 min from an ANC clinic further exacerbates the issue [50].

##### Community/Societal Factors

In remote or disadvantaged regions, particularly rural areas, accessing IPTp-SP presents significant challenges [36,51,54,65,77,89]. Long distances to health facilities exacerbate this issue, making it difficult for pregnant women to access antenatal care services and receive IPTp-SP [51,88,92]. Additionally, a lack of reliable transportation will further impede access [51]. Cultural beliefs can hinder IPTp-SP uptake, as some communities may hold views that conflict with modern medical practices, leading to hesitation in adopting new interventions [92]. Details of barriers to IPTp-SP uptake is presented in Table 4.

### 3.5. Effectiveness of IPTp-SP during Pregnancy

The effectiveness of IPTp is organised into two categories, one based on pregnancy and maternal health outcomes and effectives, and the second based on the number of doses administered.

#### 3.5.1. Effectiveness of IPTp-SP on Pregnancy and Maternal Health Outcomes

Thirteen longitudinal designs [37,40,44,46,47,58,68,96,97,98,99,100,101], 10 randomised controlled trials [69,70,102,103,104,105,106,107,108,109], 12 cross-sectional surveys [30,33,34,38,56,63,64,110,111,112,113,114], and 2 mixed-method studies [32,115] reported on the effectiveness of IPTp-SP. Across various study designs, IPTp-SP consistently reduces malaria during pregnancy [40], placental parasitemia [37,47,56,58,63,64,70,96,97,98,103,105,110], and peripheral parasitemia [106]. It is also effective in improving birth outcomes, such as increasing the birth weight in newborns of primigravida [44], reducing the number of low birth weight cases [46,47,56,63,64,68,97,103,104,112,113], and enhancing overall birth weights, particularly with the administration of three or more doses [33]. Additionally, IPTp-SP improves hemoglobin levels in primigravida [44], reduces the number of episodes of anemia and severe anemia [46,47,97,104,107], and lowers the risk of anemia in primigravida [44].

A randommised trial reported that the use of insecticide-treated nets (ITNs) further enhances IPTp-SP’s efficacy [101]. ITNs provide an additional layer of protection against mosquito bites, thereby reducing malaria transmission and complementing the preventive effects of IPTp-SP. Notably, monthly doses of IPTp-SP are more efficacious than two doses, especially among HIV-positive pregnant women [69]. Despite some contrary evidence from randomised controlled trials (RCTs) suggesting an increased risk of placental parasitemia and clinical malaria [99,108], the overall synthesis indicates that IPTp-SP is highly effective. IPTp-SP is also effective at reducing the number of cases of asymptomatic malaria during pregnancy and low birth weight [109]. Mixed-method studies and cross-sectional surveys align with these findings, highlighting reductions in maternal anemia [32,112,115], maternal parasitemia [34,111,115], and infections in newborns [64]. These studies collectively shows IPTp-SP’s crucial role in enhancing maternal and neonatal health by reducing malaria-related complications during pregnancy. See details in Table 5.

#### 3.5.2. Effectiveness of IPTp-SP Based on Doses

Nineteen (19) studies reported on the effectiveness of IPTp-SP based on the number of doses administered. For instance, a study has shown that HIV-positive mothers receiving a single dose of SP had lower levels of peripheral antibodies against apical membrane antigen-1 and variant surface antigens, as well as lower levels of cord antibodies against erythrocyte-binding antigen-175 and the parasite lysate, than HIV-positive placebo recipients [121]. Additionally, receiving at least one or more doses of IPTp-SP correlates with a decrease in placental parasitemia, indicating its efficacy in preventing malaria transmission to the fetus [37]. Moreover, receiving at least two doses has shown benefits, with a low prevalence of placental parasitemia observed among HIV-negative women [69]. The completion of at least two or three doses of IPTp-SP has also been linked to reduced placental malaria, as well as protection against low birth weights and preterm births [103]. Pregnant women receiving at least two or more doses of IPTp-SP exhibit reduced placental malaria parasitemia [56], improved birth outcomes [38], and lower odds of a low birth weight [34,56]. However, taking monthly doses of IPTp has demonstrated superiority over two doses, particularly among HIV-positive pregnant women [69]. Other evidence suggests no superiority of only two doses of IPTp-SP in HIV-positive women concerning placental malaria, anaemia, or birth outcomes [122].

Receiving at least three doses of IPTp has been associated with a decreased risk of infant low birth weight [63] and placental malaria infection [63,110]. Furthermore, pregnant women who received at least three doses showed a lower prevalence of peripheral malaria [110] and peripheral parasitemia [106]. Furthermore, receiving at least three or more doses of IPTp has shown promising results, including a reduction in malaria during pregnancy [40,104], a lower prevalence of malaria [118], and improved birth weights [33,104]. Thus, receiving at least three or more doses has been associated with an average birth weight of more than 360 g [60] and an 83% decrease in the risk of a low birth weight [117]. For individuals receiving at least four or more doses of IPTp, the benefits include lower placental infection rates [30], decreased episodes of malaria at delivery [46], and a reduced prevalence of anaemia in pregnant women [46], with no reported cases of jaundice [46]. See details in Table 6.

### 3.6. Adverse Effects of IPTp-SP

Thirteen studies reported on adverse effects of IPTp-SP [30,32,57,81,92,93,101,106,119,121,123,124,125]. Studies have linked IPTp-SP usage with increased rates of prematurity [30,57,106,123], low birth weights [30,57,106,123], and small-for-gestational-age infants [123]. In terms of intrauterine, IPTp-SP has been associated with an increased risk of miscarriages [30], congenital abnormalities [123], and abortions [119]. Furthermore, infants exposed to IPTp-SP may face extrauterine complications such as death [119], developmental delays, chronic health conditions, impaired immune function, and a heightened risk for malaria [92]. Additionally, stillbirths have been reported [119,123], along with symptoms like fever, cough, and conjunctivitis [122]. Others include dizziness [81], nausea [32,81,101], vomiting [32,101,124], skin reactions [93,124], general body weakness [32,93,101], headache [101], palpitations [32], and itching [32]. Also, studies have reported severe side effects including the emergence of PfdhFr C59R mutation [123], maternal anaemia [106], maternal death, and perinatal death [123]. Details of extracted data for this review is presented in Supplementary Materials.

## 4. Discussion

### 4.1. Summary of Findings

The findings from this review highlight the effectiveness and uptake of IPTp-SP as well as factors influencing IPTp-SP uptake. In terms of uptake, studies reported varying rates ranging from as low as 5.3% to as high as 98.9%, depending on the number of doses received. The effectiveness of IPTp-SP is supported by evidence from longitudinal studies, RCTs, cross-sectional surveys, and mixed-method studies. Longitudinal studies demonstrate its impact on reducing the number of cases of malaria during pregnancy, protecting against placental parasitemia, improving birth outcomes, and enhancing haemoglobin levels. RCTs indicate that IPTp-SP reduces the prevalence of malaria, placental malaria, and episodes of anaemia. Cross-sectional surveys reveal reductions in placental parasitemia, maternal parasitemia, and infections in newborns associated with IPTp-SP usage. Mixed-method studies further support the effectiveness of IPTp-SP by highlighting reductions in maternal anaemia and parasitemia. However, some studies suggest challenges and adverse effects associated with IPTp-SP uptake, including prematurity, low birth weights, small-for-gestational-age infants, miscarriages, congenital abnormalities, abortions, and extrauterine complications such as developmental delays, chronic health conditions, impaired immune function, and a heightened risk for malaria. Severe side effects include the emergence of specific mutations, maternal anaemia, maternal death, and perinatal death. Facilitators of IPTp-SP uptake include individual factors like education and awareness, ANC attendance, and community support, while barriers include factors such as inadequate knowledge, fear of side effects, late ANC initiation, healthcare system challenges, and cultural beliefs. 

### 4.2. Uptake of IPTp-SP

The reported rates of IPTp-SP uptake based on the number of doses received demonstrate considerable variability across different studies, indicating varying levels of adherence to the recommended regimen. The findings suggest that, while some pregnant women receive the minimum required dose, a substantial proportion still do not achieve optimal uptake levels. The low uptake rates for one dose or more, ranging from 5.3% to 98.9%, indicate significant disparities in IPTp-SP administration among pregnant women in different settings. This variability could be attributed to several factors, including differences in healthcare access, awareness about IPTp-SP, cultural beliefs, and healthcare provider practices [30,31]. For instance, higher uptake rates might be observed in areas with better healthcare infrastructure, greater community awareness programs, and effective ANC services [54].

The wide variability in uptake rates of IPTp-SP underscores the complex interplay of factors influencing pregnant women’s adherence to the recommended dosage regimen. Factors such as ANC attendance, the quality of healthcare services, patient education, and socio-economic status are pivotal in shaping IPTp-SP uptake [32,34,35]. Disparities in ANC attendance could affect the opportunity for healthcare providers to administer IPTp-SP, thereby influencing the uptake rates [32]. Additionally, variations in the quality of ANC services, including counselling on IPTp-SP, may impact pregnant women’s understanding and acceptance of the intervention [34]. Patient education initiatives that emphasize the importance of IPTp-SP in preventing malaria during pregnancy could enhance awareness and the uptake [35]. Moreover, socio-economic factors such as the income level and access to healthcare resources may influence women’s ability to attend ANC visits regularly and afford IPTp-SP, thus affecting the uptake rates [35].

The relatively low uptake rates for at least four doses or more suggest that many pregnant women do not complete the full course of IPTp-SP as recommended by the guidelines. This incomplete adherence to the recommended regimen could compromise the effectiveness of IPTp-SP in preventing malaria during pregnancy and improving maternal and neonatal health outcomes. Possible reasons for suboptimal uptake rates across different dose categories may include challenges such as stockouts of SP, inadequate ANC attendance, lack of awareness about the benefits of IPTp-SP, fear of side effects, and cultural beliefs surrounding pregnancy and medication use [30,36].

### 4.3. Facilitators and Barriers to IPTp-SP Uptake in Africa

#### 4.3.1. Facilitators

Facilitators such as education, awareness, ANC attendance, and community sensitization efforts significantly enhance IPTp-SP uptake. Higher levels of education among pregnant women and their partners correlate with better knowledge and understanding of malaria-prevention strategies, including IPTp-SP [71,73,84]. This can be attributed to the fact that education often correlates with better access to information and healthcare services, empowering individuals to make informed decisions about their health. Comprehensive knowledge about IPTp-SP and awareness of malaria risks during pregnancy promote informed decision-making and encourage pregnant women to utilize IPTp-SP [31,59]. When women understand the benefits of IPTp-SP and its role in preventing malaria-related complications during pregnancy, they are more likely to seek and adhere to the recommended doses [73].

The early initiation of ANC and completion of ANC visits are crucial facilitators of IPTp-SP uptake, emphasizing the importance of timely and consistent access to healthcare services during pregnancy [59,61]. ANC visits provide opportunities for healthcare providers to educate women about IPTp-SP and ensure its timely administration, reinforcing the importance of integrating malaria prevention into routine antenatal care [59]. Community sensitization efforts play a vital role in disseminating information and fostering a supportive environment for IPTp-SP uptake [84]. By raising awareness about the importance of malaria prevention and promoting IPTp-SP uptake at the community level, these efforts help to overcome cultural barriers and encourage positive health-seeking behaviours among pregnant women.

#### 4.3.2. Barriers

Barriers to IPTp-SP uptake stem from various individual, interpersonal, institutional, and community-level factors. Inadequate knowledge about IPTp-SP, fear of side effects, and the late initiation of ANC appointments hinder its uptake by limiting pregnant women’s understanding and acceptance of the intervention [63,94]. These issues may stem from gaps in healthcare education and counselling, coupled with misinformation about IPTp-SP’s safety and efficacy [81]. Additionally, barriers like fear of side effects could be exacerbated by cultural beliefs and previous negative experiences with healthcare interventions [81]. Individual and context-specific educational interventions and comprehensive counseling during ANC visits could help address misconceptions and alleviate concerns about IPTp-SP.

Household economic constraints, limited autonomy in decision-making, and cultural beliefs further impede access to healthcare services and influence healthcare-seeking behaviours [66,72,92]. These barriers may result from socioeconomic disparities, patriarchal norms, and traditional health practices prevalent in many African communities. Addressing these issues requires targeted interventions aimed at improving economic empowerment, promoting gender equality, and sensitizing communities about the importance of modern healthcare practices.

Healthcare system challenges, including inadequate training for healthcare providers, stockouts of SP, and long waiting times at ANC clinics, pose significant barriers to IPTp-SP uptake by limiting access to services and undermining the effectiveness of malaria-prevention programs [89,91]. These challenges could stem from resource constraints, weak healthcare infrastructure, and inefficient supply chain management systems in many healthcare settings in Africa [91]. Addressing these issues requires investments in healthcare workforce training, supply chain optimization, and infrastructure improvement to ensure the effective delivery of IPTp-SP and other essential healthcare services.

Geographical remoteness, unreliable transportation, and cultural beliefs also present substantial challenges, highlighting the need for targeted interventions to address structural and systemic barriers [51,92]. Mobile health clinics, community health workers, and community engagement initiatives can help bridge the gap and ensure equitable access to IPTp-SP for women living in remote or disadvantaged areas.

### 4.4. Effectiveness of IPTp-SP

The findings from various studies underscore the dual nature of IPTp-SP (preventive and therapeutic effects), its significant effectiveness in reducing malaria during pregnancy and improving maternal and neonatal health outcomes, alongside some notable challenges that demand deeper exploration. Longitudinal studies consistently demonstrate the positive impact of IPTp-SP in reducing malaria prevalence and protecting against placental parasitemia, ultimately leading to improved birth outcomes. These findings are crucial as they provide robust evidence supporting the implementation of IPTp-SP programs in malaria-endemic regions [37,40,96,97].

The findings reported by Umeh et al. [99] and González et al. [108] necessitate a deeper understanding of the factors influencing the effectiveness of IPTp-SP. For instance, Umeh et al. [99] found no significant reduction in malaria parasitemia among pregnant women receiving IPTp-SP, suggesting potential challenges in program implementation in the African setting or the drug’s efficacy. This indicates the importance of investigating factors such as drug-resistance patterns, patient compliance, and variations in healthcare delivery that may differently affect the effectiveness of IPTp-SP across diverse settings.

Similarly, the study by González et al. [108], indicating an increased risk of placental parasitemia and clinical malaria among pregnant women receiving IPTp-SP, raises questions about the contextual factors influencing malaria transmission dynamics and the drug’s efficacy. Possible explanations for this discrepancy include differences in malaria transmission intensity, variations in drug-resistance patterns, or limitations in study design [96]. Addressing these discrepancies requires a comprehensive understanding of local epidemiological factors, drug-resistance patterns, and healthcare infrastructure to effectively tailor malaria-prevention strategies.

Furthermore, the comparative effectiveness of different dosing regimens highlighted by studies such as Mlugu et al. [109] shows the need for personalized approaches to IPTp-SP delivery based on individual patient characteristics and regional epidemiology. While monthly doses may be more effective in certain populations, such as HIV-positive pregnant women, the lack of superiority over two-dose regimens in other contexts suggests the importance of considering specific risk factors and the local malaria epidemiology when determining optimal IPTp-SP protocols [126]. These findings emphasize the need for adaptive and context-specific strategies to optimize the impact of IPTp-SP in malaria prevention during pregnancy.

### 4.5. Adverse Effects of IPTp-SP

The adverse effects associated with IPTp-SP usage, as highlighted in various studies, underscore the complexity and potential risks involved in its administration during pregnancy. The increased rates of prematurity, low birth weights, and small-for-gestational-age infants suggest potential implications for fetal growth and development [30,57,123]. These adverse outcomes may be attributed to various factors, including drug toxicity, maternal health status, and genetic predispositions [123]. Additionally, the association of IPTp-SP with miscarriages, congenital abnormalities, and abortions raises concerns about its safety and potential teratogenic effects [30,107,123]. These findings emphasize the need for careful consideration and the monitoring of pregnant women receiving IPTp-SP to mitigate the risk of adverse pregnancy outcomes.

Furthermore, the reported extrauterine complications, such as developmental delays, chronic health conditions, impaired immune function, and increased susceptibility to malaria, suggest broader health implications for both mothers and infants [92]. These complications may result from direct drug effects, drug interactions, or underlying health conditions exacerbated by IPTp-SP usage [30]. Additionally, the occurrence of stillbirths and symptoms like fever, cough, and conjunctivitis shows the importance of vigilant surveillance and the reporting of adverse events associated with IPTp-SP [122].

The spectrum of the reported side effects, including dizziness, nausea, vomiting, skin reactions, weakness, headache, palpitations, and itching, reflects the diverse physiological responses to IPTp-SP administration [32,81,93,101,124]. While some of these side effects may be transient and mild, others, such as severe anaemia and perinatal death, pose significant health risks and warrant immediate medical attention [106,123]. These adverse reactions may be influenced by individual susceptibility, drug metabolism, and underlying health conditions, highlighting the need for personalized risk assessment and monitoring during IPTp-SP administration.

Moreover, the emergence of specific mutations, such as the PfdhFr C59R mutation, associated with IPTp-SP usage raises concerns about drug resistance and the long-term efficacy of malaria-prevention strategies [125]. It is important to note that mutations in the dhps gene, which are also prevalent, are of particular concern as they further impact the effectiveness of IPTp-SP. This suggests the importance of ongoing surveillance and research to monitor drug-resistance patterns and optimize treatment regimens to combat malaria effectively.

### 4.6. Limitations

While this scoping review followed a structured framework and employed a comprehensive search strategy across multiple databases to investigate the uptake and effectiveness of IPTp-SP in African settings, several limitations are acknowledged. The review included only studies published in the English language, potentially introducing language bias and overlooking relevant literature in other languages. Additionally, there might be a publication bias, as studies with statistically significant findings or positive outcomes are more likely to be published, potentially skewing the overall conclusions. This review did not appraise the quality of the included studies, indicating a need for caution when interpreting the findings and drawing conclusions. Furthermore, the time frame of the review and the focus on African countries might have excluded valuable insights from other regions and historical contexts.

### 4.7. Implications for Policy and Practice

Based on the findings of this study, several practical implications and recommendations for policy and practice in Africa emerge. Strengthening ANC services is paramount. This involves integrating comprehensive counselling on IPTp-SP into routine antenatal care. Pregnant women should be educated about the benefits of IPTp-SP, their recommended dosing regimen, and any potential misconceptions that should be addressed. Healthcare providers need to be trained to ensure consistent and accurate information dissemination during ANC visits. Community engagement and sensitization are crucial strategies to promote IPTp-SP uptake. Implementing community-based awareness campaigns and sensitization programs can address cultural beliefs and encourage positive health-seeking behaviours among pregnant women. Engaging community leaders, traditional birth attendants, and community health workers can help effectively disseminate information. Addressing healthcare system challenges is essential. Measures should be taken to ensure the consistent availability of SP and other essential commodities at healthcare facilities. Training and capacity-building initiatives for healthcare providers can improve their knowledge and skills in IPTp-SP administration and counselling. Streamlining ANC services to reduce waiting times and enhance their overall quality of care is imperative.

Empowering women and enhancing access to ANC services are critical steps. Economic empowerment programs targeting vulnerable populations can improve their ability to afford ANC services and essential medications like SP. Promoting gender equality and women’s empowerment initiatives can enhance women’s decision-making power regarding their health and healthcare utilization. Expanding access to ANC services through mobile health clinics, community outreach programs, and innovative service-delivery models can reach women in remote or underserved areas. Tailoring interventions to local contexts is essential. Context-specific research should be conducted to understand the unique socio-cultural, economic, and healthcare system factors influencing IPTp-SP utilization in specific regions or communities. Targeted interventions should be designed and implemented to address the specific needs and challenges faced by pregnant women and healthcare providers in each context, ensuring cultural appropriateness, contextual relevance, and long-term sustainability.

### 4.8. Recommendations for Future Studies

Future reviews should consider appraising the quality of included studies to enhance the rigour and reliability of their findings, thereby enabling more robust conclusions and recommendations. Furthermore, there is a need for longitudinal studies that follow pregnant women throughout their antenatal care to assess the uptake and effectiveness of IPTp-SP over time, allowing for a better understanding of adherence patterns and outcomes. Moreover, future research should explore the impact of socio-cultural factors, healthcare system dynamics, and geographical variations on the uptake and effectiveness of IPTp-SP to inform tailored interventions and policies. Additionally, comparative effectiveness studies evaluating different dosing regimens, delivery approaches, and adjunct interventions can provide valuable insights into optimizing IPTp-SP strategies for diverse populations and settings. Finally, interdisciplinary collaborations between researchers, healthcare providers, policymakers, and community stakeholders are essential to ensure the translation of research findings into evidence-based practices and policies that effectively address the burden of malaria during pregnancy in Africa.

## 5. Conclusions

The findings of this review provide valuable insights into the uptake and effectiveness of IPTp-SP in African settings, as well as the factors influencing its utilization. The varying rates of uptake across different studies highlight the need for targeted interventions to improve adherence to the recommended dosage regimen. While IPTp-SP has demonstrated significant effectiveness in reducing malaria during pregnancy and improving maternal and neonatal health outcomes, challenges such as suboptimal uptake rates and adverse effects underscore the complexity of its implementation. Facilitators of IPTp-SP uptake, including education, awareness, ANC attendance, and community sensitization efforts, play a crucial role in promoting its uptake and should be integrated into routine antenatal care. Conversely, barriers such as inadequate knowledge, fear of side effects, healthcare system challenges, and cultural beliefs need to be addressed through targeted interventions and policy initiatives. Interdisciplinary collaborations between researchers, healthcare providers, policymakers, and community stakeholders are essential to translate research findings into evidence-based practices and policies that effectively address the burden of malaria during pregnancy in Africa. By addressing these challenges and implementing tailored interventions, we can enhance the uptake and effectiveness of IPTp-SP, ultimately improving maternal and neonatal health outcomes and reducing the burden of malaria in African communities.

## Figures and Tables

**Table 1:** Search strategy conducted in PubMed and examples of searches conducted in main databases.

Item	Planned Search Terms
#1: Search to find IPTp-SP	Intermittent Preventive Treatment in pregnancy with Sulfadoxine-Pyrimethamine (IPTp-SP) OR IPTp with SP OR SP-IPTp OR Sulfadoxine-Pyrimethamine for Intermittent Preventive Treatment in pregnancy OR Malaria prophylaxis in pregnancy with SP OR Sulfadoxine-Pyrimethamine for malaria prevention in pregnant women OR Antimalarial treatment in pregnancy with SP OR IPTp using Sulfadoxine-Pyrimethamine OR Sulfadoxine-Pyrimethamine as intermittent preventive therapy in pregnancy OR IPTp-SP regimen
#2: Search to identify uptake of IPTp-SP during pregnancy	Uptake OR Utilization OR Intake
#3: Search to identify effectiveness	Effectiveness OR Efficacy OR Potency OR Usefulness OR Effect
#4: Search to identify pregnancy	Pregnancy, OR Antenatal period OR Gestation period OR Conception OR Prenatal period
#5: Search to identify Africa	Africa OR sub-Saharan Africa OR Eastern Africa OR Western Africa OR Central Africa OR Southern Africa OR Northern Africa OR Sure, here’s a list of all countries in Africa separated by OR Algeria OR Angola OR Benin OR Botswana OR Burkina Faso OR Burundi OR Cabo Verde OR Cameroon OR Central African Republic OR Chad OR Comoros OR Democratic Republic of the Congo OR Djibouti OR Egypt OR Equatorial Guinea OR Eritrea OR Eswatini OR Ethiopia OR Gabon OR Gambia OR Ghana OR Guinea OR Guinea-Bissau OR Ivory Coast OR Kenya OR Lesotho OR Liberia OR Libya OR Madagascar OR Malawi OR Mali OR Mauritania OR Mauritius OR Morocco OR Mozambique OR Namibia OR Niger OR Nigeria OR Republic of the Congo OR Rwanda OR Sao Tome and Principe OR Senegal OR Seychelles OR Sierra Leone OR Somalia OR South Africa OR South Sudan OR Sudan OR Tanzania OR Togo OR Tunisia OR Uganda OR Zambia OR Zimbabwe
Overall search strategy	#1 AND #2 AND #4 AND #5 Not animal * (Uptake of IPTp-SP) #1 AND #3 AND #4 AND 5 Not animal * (Effectiveness of IPTp-SP)
Filters	English language; From 1 January 2000 to 13 May 2024
**Databases**	**Search Strategy**
Search in PubMed to identify uptake and effectiveness of IPTp-SP in Africa	**Uptake of IPTp-SP:** (intermittent [All Fields] AND preventive [All Fields] AND (“therapy” [Subheading] OR “therapy” [All Fields] OR “treatment” [All Fields] OR “therapeutics” [MeSH Terms] OR “therapeutics” [All Fields]) AND (“pregnancy” [MeSH Terms] OR “pregnancy” [All Fields]) AND (“fanasil, pyrimethamine drug combination” [Supplementary Concept] OR “fanasil, pyrimethamine drug combination” [All Fields] OR “sulfadoxine pyrimethamine” [All Fields])) OR IPTp-SP [All Fields] AND uptake [All Fields] AND (“pregnancy” [MeSH Terms] OR “pregnancy” [All Fields]) AND (“africa” [MeSH Terms] OR “africa” [All Fields]) **Effectiveness of IPTp-SP:** (intermittent [All Fields] AND preventive [All Fields] AND (“therapy” [Subheading] OR “therapy” [All Fields] OR “treatment” [All Fields] OR “therapeutics” [MeSH Terms] OR “therapeutics” [All Fields]) AND (“pregnancy” [MeSH Terms] OR “pregnancy” [All Fields]) AND (“fanasil, pyrimethamine drug combination” [Supplementary Concept] OR “fanasil, pyrimethamine drug combination” [All Fields] OR “sulfadoxine pyrimethamine” [All Fields])) OR IPTp-SP [All Fields] AND effectiveness [All Fields] AND (“pregnancy” [MeSH Terms] OR “pregnancy” [All Fields]) AND (“africa” [MeSH Terms] OR “africa” [All Fields])
Search in Embase to identify uptake and effectiveness of IPTp-SP in Africa	**Uptake of IPT-SP:** (‘intermittent’:ti,ab,kw AND ‘preventive’:ti,ab,kw AND (‘therapy’:sb,ti,ab,kw OR ‘therapy’:ti,ab,kw OR ‘treatment’:ti,ab,kw OR ‘therapeutics’:mh,ti,ab,kw OR ‘therapeutics’:ti,ab,kw) AND (‘pregnancy’:mh,ti,ab,kw OR ‘pregnancy’:ti,ab,kw) AND (‘fanasil, pyrimethamine drug combination’:sc,ti,ab,kw OR ‘fanasil, pyrimethamine drug combination’:ti,ab,kw OR ‘sulfadoxine pyrimethamine’:ti,ab,kw)) OR ‘IPTp-SP’:ti,ab,kw AND ‘uptake’:ti,ab,kw AND (‘pregnancy’:mh,ti,ab,kw OR ‘pregnancy’:ti,ab,kw) AND (‘africa’:mh,ti,ab,kw OR ‘africa’:ti,ab,kw) **Effectiveness of IPTp-SP:** (‘intermittent’:ti,ab,kw AND ‘preventive’:ti,ab,kw AND (‘therapy’:sb,ti,ab,kw OR ‘therapy’:ti,ab,kw OR ‘treatment’:ti,ab,kw OR ‘therapeutics’:mh,ti,ab,kw OR ‘therapeutics’:ti,ab,kw) AND (‘pregnancy’:mh,ti,ab,kw OR ‘pregnancy’:ti,ab,kw) AND (‘fanasil, pyrimethamine drug combination’:sc,ti,ab,kw OR ‘fanasil, pyrimethamine drug combination’:ti,ab,kw OR ‘sulfadoxine pyrimethamine’:ti,ab,kw)) OR ‘IPTp-SP’:ti,ab,kw AND ‘effectiveness’:ti,ab,kw AND (‘pregnancy’:mh,ti,ab,kw OR ‘pregnancy’:ti,ab,kw) AND (‘africa’:mh,ti,ab,kw OR ‘africa’:ti,ab,kw)
Search in Scopus to identify uptake and effectiveness of IPTp-SP in Africa	**Uptake of IPTp-SP:** TITLE-ABS-KEY(intermittent AND preventive AND (therapy OR treatment OR therapeutics) AND (pregnancy) AND (“fanasil, pyrimethamine drug combination” OR “sulfadoxine pyrimethamine”)) OR TITLE-ABS-KEY (IPTp-SP AND uptake AND pregnancy AND africa) **Effectiveness of IPTp-SP:** TITLE-ABS-KEY (intermittent AND preventive AND (therapy OR treatment OR therapeutics) AND (pregnancy) AND (“fanasil, pyrimethamine drug combination” OR “sulfadoxine pyrimethamine”)) OR TITLE-ABS-KEY (IPTp-SP AND effectiveness AND pregnancy AND africa)
Search in Web of science to identify uptake and effectiveness of IPTp-SP in Africa	**Uptake of IPT-p-SP:** TS = (intermittent AND preventive AND (therapy OR treatment OR therapeutics) AND (pregnancy) AND (“fanasil, pyrimethamine drug combination” OR “sulfadoxine pyrimethamine”)) OR TS = (IPTp-SP AND uptake AND pregnancy AND africa) **Effectiveness of IPTp-SP:** TS = (intermittent AND preventive AND (therapy OR treatment OR therapeutics) AND (pregnancy) AND (“fanasil, pyrimethamine drug combination” OR “sulfadoxine pyrimethamine”)) OR TS = (IPTp-SP AND effectiveness AND pregnancy AND africa)
E.g., Search in JSTOR to identify uptake and effectiveness of IPTp-SP in Africa	**Uptake of IPTp-SP:** (ti:(intermittent AND preventive AND (therapy OR treatment OR therapeutics) AND (pregnancy) AND (“fanasil, pyrimethamine drug combination” OR “sulfadoxine pyrimethamine”))) OR (ti:(IPTp-SP AND uptake AND pregnancy AND africa)) **Effectiveness of IPTp-SP:** (ti:(intermittent AND preventive AND (therapy OR treatment OR therapeutics) AND (pregnancy) AND (“fanasil, pyrimethamine drug combination” OR “sulfadoxine pyrimethamine”))) OR (ti:(IPTp-SP AND effectiveness AND pregnancy AND africa))

**Table 2:** Rate of uptake of IPTp-SP.

Dose	Rate of Uptake (%)	Sample Size	Country	Authors
At least 1 dose of IPTp-SP	5.3	1926	Ghana	[30]
	6.0	1922	Ghana	[33]
	24.0	487	Ghana	[32]
	35.8	565	Uganda	[34]
	38.0	201	Cameroon	[41]
	41.0	724	Kenya	[42]
	75.5	391	Malawi	[43]
	82.0	2232	Ghana	[44]
	84.1	1030	Gabon	[45]
	92.4	2785	Uganda	[46]
	95.3	8131; 439	Malawi; Cameroon	[47,48]
	97.7	1820	Uganda	[49]
	98.9	375	Ghana	[31]
At least 2 doses of IPTp-SP	15.0	487	Ghana	[32]
	15.5	1926	Ghana	[30]
	19.2	8131	Malawi	[47]
	21.0	724	Kenya	[42]
	31.3	500	Uganda	[50]
	44.8	500	Uganda	[50]
	54.9	439	Cameroon	[48]
	56.6	565	Uganda	[34]
	57.4	1030	Gabon	[45]
	70.2	426	Malawi	[51]
	95.5	1922	Ghana	[33]
At least 3 doses of IPTp-SP	6.0	565	Uganda	[34]
	9.0	487	Malawi	[32]
	18.0	5901	Uganda	[52]
	29.8	426	Malawi	[51]
	33.2	1926	Ghana	[30]
	34.3	6143	Gambia	[53]
	45.3	4254	Ghana	[54]
	46.5	1141	Mozambique	[55]
	47.0	465	Cameroon	[56]
	58.9	2174	Gabon	[57]
	61.4	500	Tanzania	[58]
	63.7	1021	Mali	[59]
	64.5	1922	Ghana	[33]
	66.2	692	Ghana	[60]
	80.8	375	Ghana	[31]
	93.2	8526	Sierra Leone	[35]
At least 4 doses of IPTp-SP	7.5	3686	Zambia	[36]
	39.5	375	Ghana	[31]
	42.3	1926	Ghana	[30]
At least 5 doses of IPTp-SP	17.1	375	Ghana	[31]
Full dose (as reported in studies)	8.0	4111	Tanzania	[61]
	14.5	255	Ghana	[62]
	14.5	255	Benin	[63]
	17.5	888	Cameroon	[64]
	29.5	18603	Burkina Faso, Ghana, Mali, Malawi, Kenya, Nigeria, Sierra Leone, and Uganda	[65]
	41.0	400	Nigeria	[66]
	97.0	2419	Tanzania	[67]

**Table 3:** Facilitators of IPTp-SP uptake.

Main Theme	Specific Factor	Author
**Individual**	Any level of education	[59,71,84]
	Primary or higher education	[61]
	Secondary or higher education	[34,35,52,73,74]
	No education	[53]
	Women between age 15–19	[54]
	Women aged 30–39 years	[75]
	25–29 years	[76]
	Women 15 and 24 years	[49]
	Higher age (36–39 years)	[77]
	Age more than 19 years	[84]
	Married or living with a partner	[75]
	Knowledge about IPTp-SP	[31,48,49]
	Knowledge about prophylaxis for malaria prevention	[78]
	Knowledge of the recommended dose of IPTp-SP	[59]
	Knowledge about malaria in pregnancy	[51]
	Awareness of the dangers of malaria in pregnancy	[51]
	Awareness about malaria prevention programs	[75]
	Awareness about IPTp-SP	[55]
	Low awareness of IPTp-SP	
	Attendance ANC in the first trimester	[61]
	ANC in the first or second trimester	[56,75]
	Early initiation of ANC (First or second trimester)	[59]
	Attendance at government health facilities	[61]
	3 or more ANC visits	[59]
	4 or more visits	[51,52,56,61,63]
	8 or more ANC visits	[31]
	Increase ANC visits	[85]
	Increased ANC attendance	[57]
	Having 3 or more children	[49]
	Women with advanced pregnancy (24–40 weeks)	[77]
	Secundigavida	[72]
	Having 3 or more children	[49]
	Parity 0–3	[53]
	Women who tested positive for malaria	[77]
	Anaemic pregnant women l	[77]
	Use of ITN	[68]
**Interpersonal**	Husband’s education	[74]
	Richer households	[34,65,71]
	Education of man on dangers of malaria in pregnancy	[70]
	Education and counselling on malaria and ANC attendance	[79]
**Institutional**	Proximity of health facility	[51]
	Trust in the healthcare system	[80]
	Continuous training of HCPs to improve their knowledge	[70]
	High stock levels	[62,63]
	Distribution of IPT-SP at ANC	[69,74]
	Exposure to media messages	[36,37,53,56,71]
	Utilising DOTS strategy	[81]
	Wider coverage of ANC	[80]
	≥8 ANC visits	[31]
	4 ANC visits	[80]
	Increased ANC attendance	[35,45,57]
	Seen by skilled attendant	[49]
	Nurses persuade mothers to take SP	[81]
	Supervision of HCPs	[70]
**Community/Societal**	Rural dwelling	[50]
	Residence	[53]
	Urban dwellers	[82]
	Being a resident in an urban area	[77]
	Encouragement of social networks	[80]
	Favourable social norms for IPTp-SP	[83]
	Community sensitisation	[72]
	Cost of IPTp-SP delivery	[46]
	Community delivery/distribution of IPTp-SP	[79,86]
	High resistance areas	[68]
	Presence of health centres	[59]
	Distance from the nearest health centre (0–5 km)	[72]

**Table 4:** Barriers to IPTp-SP uptake.

Main Theme	Specific Factor	Author
**Individual**	Poor knowledge about IPTp-SP	[95]
	Women who had higher knowledge about malaria prevention were less likely to take adequate IPTp-SP	[36]
	Inadequate knowledge of malaria	[50]
	Low knowledge on IPTp-SP services	[89]
	Low awareness of IPTp-SP	[55,82,89]
	Fear of side effect	[81]
	The perception that IPTp-SP has adverse effect on pregnant women	[93]
	Refusal to take SP	[80]
	Late enrolment	[94]
	Skipping ANC appointment	[80]
	Initiating ANC attendance late	[80]
	Irregular ANC attendance	[85]
	At least three ANC visit	[35]
	Late 1st ANC visit	[81]
	Lack of ANC attendance	[32]
	Receiving first dose in the third trimester	[63]
	Multigravida women are less likely to complete the recommended dose	[43]
	Higher parity of more than four	[35]
	Higher parity	[76]
	At least one delivery	[76]
	Pregnant woman testing HIV positive	[81]
	History of child death	[42]
	Pregnant woman presenting with malaria	[81]
	Women with less than 8 years of education	[42]
	Women with less than 8 years of education	[42]
	No formal education	[76]
	Having no or only primary education	[55]
	Lower educational attainment	[50]
	Poor women	[54]
	Being single	[42]
**Interpersonal**	Least poor households	[84]
	Richer Households	[65]
	Lack of autonomy or freedom to receive IPTp-SP	[66]
**Institutional**	Lack of training and supervision for health care workers	[91]
	Inadequate information provided to women on IPTp-SP	[48,80,85]
	Inadequacy of routine training of HCP	[48]
	HCP refusing services to women	[91]
	Frequent absences and staffing changes mandated on the job	[90]
	HCP uncertainty about the safety and efficacy of IPTp-SP	[91]
	Inconsistent provision of ANC,	[91]
	Perception that IPTp has adverse pregnant women	[93]
	Inadequate counselling or support for pregnant women	[92]
	Cost of ANC	[80]
	Health financing challenges	[77]
	HCP absenteeism	[51]
	User fees charged by HCW	[88]
	HCW ask for bribes from clients	[88]
	Not being offered IPTp-SP	[80,83,85]
	Frequent stock out	[57,66,82,89,92]
	Inadequate information provided to women on IPTp-SP	[48,80,85]
	Misinformation standard doses	[88]
	Lack of comprehensive knowledge on IPTP policy	[77]
	Inadequate supply of SP to the health sector	[91]
	Demand and supply bottlenecks	[77]
	Inadequate supply for SP for private sector	[91]
	Limited healthcare infrastructure	[92]
	Long waiting time	[89,92]
	Poor implementations of DOTs	[66]
	Non-institutional births	[55]
	Inconsistent guidelines for IPTP	[91]
	Inadequate guidelines for HCP	[91]
	More than 30 min working distance to the ANC clinic	[50]
**Community/Societal**	Remote region/disadvantaged region/rural area	[36,51,54,76,77,89]
	Long distance to Health facilities	[51,88,92]
	Lack of transport	[51]
	Cultural beliefs	[92]

**Table 5:** Effectiveness of IPTp based on study design.

Design	What Indicates Effectiveness
Longitudinal studies	Reduction in malaria in pregnancy [40]
	Protection or reduction in placental parasitaemia [37,47,58,96,97]
	3-fold decrease in placental parasitaemia [98]
	Did not significantly reduce prevalence of malaria parasitaemia [99]
	Improved birth outcome [100]
	Fewer low birth weight [46,47,68,97]
	Greater birth weight in new borns of primigravida [44]
	Improved haemoglobin levels of primigravida [44]
	Fewer episodes of anaemia & severe anaemia [47,97,107]
	Low risk of anaemia of primigravida [44]
	Increase in maternal haemoglobin level [47]
	ITN was important in determining efficacy of IPTp [101]
Cross-sectional studies	Reduction in placental parasitaemia [30,35,56,63,64,110]
	Lower odds of maternal parasitaemia [34,111]
	Reduced infections in new-borns [64]
	Lower odds for infant low birth weight [34,56,63,64,112,113]
	Low cord malaria [110]
	Improved birth weight in those with 3 or more doses [33]
	No difference in terms of LBW, placental malaria, and malaria infection [101]
	Low risk of maternal anaemia [34,64,114]
	Reduction of maternal anaemia in primigravid woman [41]
	Higher haemoglobin levels [111]
	Prophylactic effect against maternal anaemia at delivery [112]
	Paucigravida protective against low birth weight [38]
	Reduced placental infection [38,116]
	Multigravida reduced incidence of preterm delivery [38]
	Improved birth outcomes [38] Average birth weight more than 360 g [60]
	83% decrease in risk of low birth weight [117]
	Less risk of malaria [110,114]
	Lower prevalence of malaria in pregnancy compared to only ITN or those with no intervention [118]
	Parasite density for all women decreased after the first dose but no further reduction was seen after the 2nd dose [119]
Randomised controlled trial	Infants had lower prevalence of malaria [102]
	Protection against LBW [103,104]
	Protection against preterm births [103]
	Monthly doses were efficacious than 2 doses during pregnancy in HIV positive pregnant women [69]
	Reduced placental malaria [70,103,104,105]
	Increased risk of placental parasitaemia [108]
	Increased clinical malaria [108]
	HIV negative women had low prevalence of placental parasitaemia [69,120]
	Reduction in antibodies in HIV infected women [121]
	Reduction in peripheral parasitaemia [120]
	Peripheral parasitaemia were less common in those with 3 doses [106]
	Reduction of risk of malaria infections in primigravida & secundigravida women [72]
	Decreased episodes of malaria at [107]
	Decreased prevalence of anaemia in women who received [104,119]
	Reduction in probability of anaemia at delivery [105]
	No prevalence of jaundice [119]
	Higher levels of haemoglobin [119]
	Reduction in probability of low birth weight [105]
	No evidence that 2 doses only was superior to IPTp in HIV + women in terms of placental malaria, anaemia, or birth outcomes [122]
	Not associated with reduced maternal malaria [112]
	Compared to IPTp-sp, IPTp-DHP was more effective in reducing risk for asymptomatic malaria during pregnancy and LBW [109]
	No significant reduction in episodes of clinical malaria [67]
Mixed-method studies	Reduction of maternal anaemia [32,115]
	Reduction of maternal parasitaemia [115]

**Table 6:** Effectiveness of IPTp based on doses.

Doses	What Indicates Effectiveness
At least 1 dose	Reduction in antibodies in HIV infection women [121] (Novel evidence)
	Reduction in placental parasitaemia [37]
Only 2 doses	Monthly doses were efficacious than 2 doses during pregnancy in HIV positive pregnant women [69]
	Among HIV negative had low prevalence of placental paracetamol [69]
At last 2 or more doses	Reduced placental malaria [103]
	Protection against LBW [103]
	Protection against preterm births [103]
	Reduction in placental malaria parasitaemia [34,38,56]
	Improved birth outcomes [38]
	Lower odds for low birth weight [34,56]
	Paucigravida protective against low birth weight [38]
	Multigravida reduced incidence of preterm delivery [38]
	Reduced of risk of malaria infections in primigravida & secundigravida [72]
	Lower odds of maternal parasitaemia [34]
At least 3 or more doses	Decreased risk of infant with LBW [63]
	Decreased risk of placental malaria infection [63,110]
	Low venous/peripheral malaria [110]
	Peripheral parasitaemia were less common in those with 3 doses [110]
	Low cord malaria [110]
	No evidence that 2 doses only was superior to IPTp in HIV + women in terms of placental malaria, anaemia, or birth outcomes [122]
	Reduction in malaria in pregnancy [40,104]
	prevalence of malaria in pregnancy compared to only ITN or those with no intervention [118]
	Average birth weight more than 360 g [60]
	83% decrease in risk of low birth weight [117]
	Improved birth weight [33,104]
	Reduction in Anaemia [104]
At least 4 or more doses	Had lower placental infection [30]
	Decreased episodes of malaria at delivery [119]
	Decreased prevalence of anaemia in pregnant women [119]
	No prevalence of jaundice [107]

## Data Availability

The raw and analysed data are available with the corresponding author on reasonable request.

## Supplementary Materials

The following are available online at https://www.mdpi.com/article/10.3390/diseases12090203/s1, Extracted Data [30,31,32,33,34,35,36,37,38,39,40,41,42,43,44,45,46,47,48,49,50,51,52,53,54,55,56,57,58,59,60,61,62,63,64,65,66,67,68,69,70,71,72,73,74,75,76,77,78,79,80,81,82,83,84,85,86,87,88,89,90,91,92,93,94,95,96,97,98,99,100,101,102,103,104,105,106,107,108,109,110,111,112,113,114,115,116,117,118,119,120,121,122,123,124,125,127,128,129,130,131].
