# Supplementary material for: Uptake and Effectiveness of Intermittent Preventive Treatment with Sulfadoxine-Pyrimethamine during Pregnancy in Africa: A Scoping Review
Source: Diseases. 2024 Sep 4;12(9):203. doi: 10.3390/diseases12090203 (PMC11431087; doi:10.3390/diseases12090203)
Supplement: Supplementary file 1 [file diseases-12-00203-s001.zip › diseases-3107520-supplementary.pdf]

Supplementary Materials:

Extracted data

Author(s) & Year/ Country	Study Purpose	Design	Population	Sample size	Rate of IPTp-SP	Facilitators	Barriers to access to IPTp-SP	Effective-ness of IPTp-SP	Adverse effects	
									Mothers	Foetus
[37]<br><br>Burkina Faso	To assess the disease burden after imple-menting IPTp-SP	Longitudi-nal/ cohort	Women in their third trimester and at 15 years or more and had placen-tas available	2014	ANC = 96.2% and Delivery unit = 93.5%			≥1, 2 and 3 doses<br><br>of IPTp/SP were asso-ciated with a re-duction in the preva-lence of placental parasi-taemia		
[94]<br><br>Malawi	To examine women's knowledge and perceptions about the use of medication in pregnancy and the timing and motivation con-cerning use of antenatal clinic services.	Mixed method	Pregnant women	248			Unclear mes-sages about IPT-SP from nurses; Poor timing of SP-1; periodic shortages of SP; women's limited understanding of IPT- SP; tendency for late enrol-ment; and nurses' under-performance.			

[80]<br><br>Ghana	To explore the barriers to the uptake of IPTp-SP	Qualitative study	Healthcare providers, pregnant women, opinion leaders, Facility managers etc,	74		Trust in the healthcare system and wider coverage of ANC services, Socio-cultural factor such as encouragement from social networks influenced utilization of ANC services and IPTp-SP uptake.	Inadequate information provided to<br><br>women accessing ANC, stock-outs and fees charged for ANC services reduced access to<br><br>IPTp-SP. Individual factors such as refusing to take<br><br>SP, skipping ANC appointments and initiating ANC attendance late affected uptake.			
[95]<br><br>Nigeria	To determine the effects of training primary health care workers on the utilization of IPTp	Quasi-Experiment	Pregnant women who attended ANC	170	68%		Poor knowledge about IPTp-SP			
[127]<br><br>Tanzania	To appraise the prevalence of malaria and anaemia in antenatal mothers; and explore the factors	Cross-sectional survey	Pregnant mothers on routine ANC visits	395	60%		Lack of awareness on the timing for IPTp-SP			

	influencing coverage of<br><br>IPTp-SP under operational conditions in the national programme for<br><br>malaria control in pregnancy.									
[71]<br><br>Nigeria	To explore the differentials IPTp uptake	Cross-sectional survey	Women who had ever given birth.	24,344	76%	Women who<br><br>have access to media use, married, educated and non-poor were more likely to uptake<br><br>IPTp.				
[48]<br><br>Cameroon	To assess factors<br><br>associated with the uptake of IPTp 9among pregnant women attending ANCs.	Cross-sectional survey	Healthcare workers and pregnant women.	439	95.3% (one dose) and 54.9% (three doses).	Better knowledge on IPTp-SP	Medication stock out and inadequacy of routine trainings for the health providers.			
[85]<br><br>Uganda	To explore access to IPTp-SP	Cross-sectional survey	Women who have given birth.	453	52.3% (two or three doses)	Increased ANC visits.	Not given by midwife for unknown reason, SP stock-outs and irregular ANC attendance.			

[86]<br><br>Malawi	To examine the effectiveness of IPTp-SP	Longitudinal/ cohort	Women without HIV/AIDS	703				IPTp-SP was associated with improved<br><br>Birth outcomes.		
[62]<br><br>Ghana	To determine the level of uptake of IPTp-SP.	Cross-sectional survey	12 weeks postnatal women seeking care.	255	Full dose (14.5%).	Four ANC visits or more and high stock levels.	Receiving first dose in the third trimester.			
[55]<br><br>Mozambique	To access IPTp-SP coverage and associated factors.	Cross-sectional survey	Mothers delivering.	1,141	Three or more dose (46.5%).		Non-institutional deliveries, first ANC visit after week 28, low awareness of IPTp-SP, having no or only primary education and rural residents			
[83]<br><br>Madagascar	To explore the uptake and determinants of IPTp-SP.	Cross-sectional survey	Mothers with children below 2 years.	910	Two doses or more (11.7%).	High knowledge, positive attitudes, exposure to malaria messages and favourable social norms for IPTp-SP.	Not being offered IPTp-SP and low ANC attendance.			

[128] Nigeria	To determine the effectiveness of IPTp-SP.	RCT	Women receiving ANC sessions.	459				Compared to IPTp-SP, ISTp-AL had low parasitaemia, low level of low birthweight and few complaints of fever.		
[52] Uganda	To assess the prevalence and associated factors for IPTp-SP uptake.	Cross-sectional survey	Women who had live birth prior to data collection	5,901	Three doses or more (18%).	Attained secondary education, attended ANC 4 or more times, and exposed to radio messages.	Age 34 years or more and attending first ANC in third trimester.			
[56] Cameroon	To assess the IPTp-SP coverage and its effectiveness.	Cross-sectional survey	Parturient women	465	Three doses or more (47%).	Attending ANC 4 or more times and attending ANC in first and second trimester.		Taking 3 or more dose was associated with increased placental parasitaemia. Taking 2, 3 or more doses was associated lower odds for		

								low birth weight.		
[87]<br><br>Burkina Faso	To examine the delivery of IPTp-SP.	RCT	Women attending ANC care.	744		Community delivery of IPTp-SP led to a greater IPTp doses with no apparent decrease in ANC attendance.				
[61]<br><br>Tanzania	To explore the predictors of optimal doses of IPTp-SP.	Cross-sectional survey	Women who had 2 or less years of live birth.	4,111	8% had optimum uptake.	Primary and secondary or higher education, attending ANC in first trimester, attending 4 or more ANC visits, and attending government health facilities.				
[59]<br><br>Mali	To explore the factors associated with IPTp-SP adherence.	Cross-sectional survey	Village women with 2 years live birth.	1,021	3 or more doses (63.7%).	Attending 3 or more ANC, early initiation of ANC (first or second trimester), presence of health centre, ability to read, knowledge of utility of the drug and knowledge of recommended dose of IPTp-SP.				

[60]<br><br>Ghana	To explore determinants of IPTp-SP in rural Ghana.	Cross-sectional survey	Pregnant women attending ANC in village.	692	3 or more dose at delivery (66.2%).			3 or more dose is associated with average birth weight increase of more than 360g.		
[54]<br><br>Ghana	To explore uptake of IPTp-SP and determinants.	Cross-sectional survey	Women with 2 or less live birth.	4,254	3 or more dose (45.3%)		Women between age (15-19) years, poor women and remote region or disadvantage region.			
[51]<br><br>Malawi	To assess determinants of IPTp-SP uptake during pregnancy among postpartum women	cross-sectional survey.	postpartum women	426	29.8%, - 3> doses<br><br>70.2% - 2 or less	***Individual factors***<br><br>• At least four ANC visits<br><br>• knowledgeable about malaria transmission<br><br>• Awareness of dangers of malaria	***Structural factors:***<br><br>• SP not taking under DOTs.<br><br>• Long distance to health facility/lack of transport.<br><br>• health provider absenteeism<br><br>• Health facility located in rural areas	-	-	-

						in pregnancy<br><br>***Cultural characteristics:***<br><br>• Problems with /limited spousal escort to ANC<br><br>• Reduced need for spousal permission to attend ANC.<br><br>• 1st ANC attendance in 1st trimester<br><br>***Structural factors***<br><br>• Use of DOTS for SP<br><br>• Proximity of health facility.				
[117]<br><br>Tanzania	To determine the prevalence of adverse birth outcomes as well as the associated risk	Cross-sectional-study	Pregnant women	631	97.8%	-	-	≥3 doses of IPTp-SP were associated with 83% decrease	-	-

	factors, among pregnant women who received IPTp-SP in a region with low malaria prevalence.							in risk of Low birth weight (LBW).		
[31]<br><br>Ghana	To determine the factors influencing adherence to the new IPTp-SP policy	Cross-sectional	Nursing mothers	375	• 98.9% - 1 dose<br>• 95.5% - 2 doses<br>• 80.8% -3 doses<br>• 39.5% - 4 doses<br>• 17.1% - 5 doses	≥8 ANC visits<br><br>knowledge of IPTp-SP	-	-	-	-
[74]<br><br>Nigeria	To determine the predictors of IPTp uptake in the last pregnancy among women	Cross-sectional study (community-based)	Women of reproductive age	340	74.2%	• Husband education at least secondary level or higher	-	-	-	-

[75]<br><br>Tanzania	To identify factors that are responsible for the uptake of IPTp among pregnant Tanzanian women	Cross-sectional study	Women aged 15–49 years who had a live birth.	1,616		30–39 years, Married or living with partner, First or second pregnancy.<br>• First antenatal visit in the first or second trimester.<br>• Awareness about malaria prevention programs<br>• Distribution of IPT at ANC	• Younger or older than 30-39<br>• Never married or divorced/separated.<br>• Two or more children<br>• First antenatal visit in the third trimester<br>• Self-employment<br>• Low income	-	-	-

[76]<br><br>Ivory Coast	To investigate the association between antenatal care visits and the uptake of tetanus toxoid and intermittent preventive treatment for malaria during pregnancy in Ivory Coast.	Cross-sectional-design	Pregnant women	946	17.83%.	• Age of women (25-29 years)<br>• Residence in rural area<br>• No formal education<br>• Had at least one delivery<br>• At least 3 ANC visits	-	-	-	-
[81]<br><br>Malawi	To examine the experiences and perspectives of health care providers on the provision of IPTp for malaria during ANC and to identify potential barriers and facilitators to the delivery of IPTp.	Qualitative	Nurses & midwives	46	-	• Nurses persuade mothers to take SP.<br>• DOTS strategy	• Late 1st ANC 1st visit<br>• Fear of side effects<br>• Pregnant woman testing HIV+<br>• Pregnant woman presenting with malaria<br>***Women's objections about SP***	-	• Dizziness<br>• Nausea	-

							• SP tablet is hard & large to swallow.<br>• SP leaves bitter taste.<br>• Can't take SP on empty stomach.<br>• Lack of understanding of prophylaxis			
[38]<br>Zambia	To evaluate the effectiveness of sulphadoxine-pyrimethamine for intermittent preventive treatment of malaria in pregnancy	Cross-sectional survey	Pregnant women attending ANC	387	91.2%	-	-	***Pauci-gravids***<br>2 or > doses of SP-IPTp were associated with<br>protective effect on low birth weight and<br>placental infection ***Multi-gravids***	-	-

								significantly protective for<br><br>preterm delivery and<br><br>Improved birth outcome		
[102]<br><br>Ghana	To compare the incidence of malaria in infants born to mothers who received either IPTp-SP or ISTp-AL during their pregnancy.	RCT	Pregnant women attending ANC	988	-	-	-	Children born to women in IPTp-SP had lower incidence rate of clinical malaria	-	-
[118]<br><br>Cameroon	To assess the usage and effectiveness of IPTp-SP and ITNs on malaria in pregnancy.	Cross-sectional hospital-based	Pregnant women attending ANC	410	63.2%	-	-	IPTp-SP is effective in reducing malaria prevalence in pregnancy.<br><br>women using only IPT-SP have least malaria	-	-

								prevalence (7.70 %) with three successive doses of SP compared to those using ITN only or no intervention.		
[91]<br><br>Uganda	To identify and assess the challenges that impede IPTp uptake.	Qualitative	Pregnant women attending ANC, health workers, district health officials, and opinion leaders.	**46**	-	-	• Inconsistent guidelines for IPTp provision<br>• Inadequate knowledge of guidelines by health workers<br>• Lack of training and supervision opportunities for health workers<br>• health workers uncertainty about the safety and efficacy of SP	-	-	-

							• Inadequate supply of SP to the private sector<br>• Inconsistent provision of ANC, leading to missed opportunities for the provision of IPTp<br>• Negative impact of health workers refusing services to women attending ANC<br>• Supply-side issues, such as Stock-outs of SP in public facilities			
[49]<br>Uganda	To examine the factors associated with taking two or more doses of therapy among women who had had the	Cross-sectional survey	Women who had had the most recent live birth	1820	97.71 % one dose<br>45.16 % - 2 or more doses	• Knowledgeable regarding SP<br>• Seen by a skilled attendant at ANC	• Women older than 35 years	-	-	-

	most recent live birth					• Having three or more children<br>• Women 15 and 24 years				
[125]<br>Burkina Faso	To explore the relationship between IPTp-SP and the presence of mutant parasites.	Cross-sectional survey	Pregnant women	109		•	•	-	*Recent* uptake of IPTp-SP was at higher odds of both the *Pfdhfr* C59R mutation	-
[116]<br>Mali	To investigate factors associated with taking 3 doses or less of IPTp-SP	Cross sectional survey	Pregnant women<br>(15-49)	7,758		Accessibility to media (listening to radio)	Age less than 20 years was associated with lower than 3 doses	SP was efficacious for the prevention of placental malaria		
[69]<br>Malawi	To compare the efficacy of monthly IPTp-SP with a 2-dose regimen for the prevention of placental parasitaemia in HIV-positive and -negative women	RCT	Pregnant HIV positive and negative women	698		Providing SP at all ANC visits		Monthly IPTp-SP was more efficacious than a 2-dose regimen in HIV-positive pregnant women.		

								HIV negative women who received monthly SP had extremely low prevalence of placental parasitaemia.		
[106] Zambia	To compare the standard 2-dose SP IPTp regimen to the intensive monthly IPTp among HIV-positive Zambian women	RCT	HIV positive pregnant women in their second or third trimesters of pregnancy	456				Peripheral parasitaemia and anaemia were less common in pregnant women who received 3 doses of SP than 1or 2 doses.	Single-dose SP was associated with higher proportions of maternal anaemia, peripheral and cord blood parasitaemia	Low doses of SP were significantly associated with infant prematurity, and low birth weight
[122] Zambia	To compare the standard 2-dose SP regimen with	RCT	HIV positive pregnant women	456				No evidence that intensive monthly		

	monthly IPTp among a cohort of HIV-positive pregnant women		between 16 and 28 weeks of gestation					dosing was superior to standard dosing of IPTp-SP in HIV positive women in terms of placental malaria, maternal anaemia, or birth outcomes.		
[120] Mali	To evaluate the efficacy and safety of 2 IPT regimens compared with the standard of care in preventing the adverse effects of malaria in primigravid and secundigravid women.	RCT	Pregnant women in their first or second pregnancy and between 16 and 26 weeks of gestation, attending ANC	1163 women				Women who gave birth in the IPT/CQ and weekly CQ groups were more likely to have peripheral and placental malaria, compared with women who gave birth in		neonates and infants 6 weeks of age were cough (21.0%), fever (13.2%), conjunctivitis (4.8%), and skin infection (3.4%),

								the IPTp-SP group		
[112]<br><br>Democratic Republic of Congo	To evaluate the prophylactic effects of IPTp-SP on maternal malaria and adverse pregnancy outcomes	Cross-sectional study	Pregnant women and their newborns	844				IPTp-SP was not associated with reduced maternal malaria.<br><br>Increased doses of SP uptake had a significant effect against low birth weight.<br><br>Increased doses of SP uptake during pregnancy had a significant prophylactic effect against maternal anaemia at delivery		

[77]<br><br>Ghana	To examine the effect of individual, household, and health-related factors on the uptake of optimal doses of IPTp-SP	Cross-sectional survey	Pregnant women within the reproductive age range of 15-49 years	5181	Optimal uptake of IPTp-SP (SP≥ 3 dose) for malaria in pregnancy was low	Being resident in an urban area<br><br>Higher age (36-39)<br><br>Women with advanced pregnancy (24-40 weeks) took optimal doses of IPTp.<br><br>Women who tested positive for malaria received optimal SP doses.<br><br>Pregnant women who were anaemic were more likely to take optimal doses	Rural dwelling<br><br>Lower age (15-24 years<br><br>Pregnant women (16-20 weeks) took lower doses of IPTp.<br><br>Demand and supply bottlenecks,<br><br>health providers' lack of comprehensive knowledge<br><br>on IPTp policy<br><br>Health financing<br><br>Pregnant women who were not anaemic were less likely to take sub-optimal doses of SP			
[107]<br><br>Uganda	To assess whether traditional birth attendants,<br><br>drug-shop vendors,	Longitudinal/ Cohort	Pregnant women	2,785	67.5% (pregnant women receiving community care) and 39.9% (receiving care at health centre).		The cost per woman receiving the full course of IPTp was higher when delivered via community care at US$2.60	Women receiving community-<br><br>based care had fewer episodes		

	community reproductive health workers and adolescent peer mobilisers could administer IPTp-SP						compared with US$2.30 at health centres,	of anaemia or severe anaemia and fewer low birth weight babies.		
[46] Uganda	To assess the impact of a community-based delivery system of IPT for malaria in pregnancy with SP on access, parasitaemia, anaemia and low birth weight	RCT		2785				Parasite density for all women decreased after the first dose of SP, but no further reduction was seen after the second dose		
[57] Gabon	To determine the factors influencing antenatal care (ANC) visit attendance, complete intermittent preventive	Cross-sectional study	pregnant women	2,174	58.87% completed at least 3 doses	Increased ANC attendance was directly proportional to IPTp-SP completion.	IPTp-SP stock shortage in prenatal counselling services<br><br>Lack of money to purchase IPTp-SP treatment			1. Low birth weight<br><br>2. Premature birth

	treatment with sulfadoxine-pyrimethamine (IPTp-SP) and its impact on the health of pregnant women and their newborn babies living in semi-urban and rural areas of south-eastern Gabon.									
[73]<br><br>Mozambique<br><br>Nigeria<br><br>Sierra Leone<br><br>Tanzania<br><br>Uganda<br><br>Burkina<br><br>Ghana<br><br>Kenya	To examine the factors associated with the uptake of IPTp-SP among pregnant women in sub-Saharan Africa.	Cross-sectional survey	pregnant women	43,961	Mozambique = 41.91%<br><br>Nigeria = 20.82%<br><br>Sierra Leone = 30.61%<br><br>Tanzania = 22.52%<br><br>Uganda = 39.59%<br><br>Burkina = 57.46%<br><br>Ghana = 59.64%	1. Women aged 40-44 years were more likely to complete the recommended doses<br><br>2. Secondary/higher education had a higher chance of completing the doses<br><br>3. Exposure to malaria messages via television, and radio				

Liberia<br>Madagascar<br>Mali<br>Malawi					Kenya = 21.95%<br>Liberia = 23.57%<br>Madagascar = 10.08%<br>Mali = 22.26%<br>Malawi = 34.65					
[33]<br>Ghana	To assess the coverage achieved by the new policy and its impact on maternal and neonatal birth outcomes	Cross-sectional study	Pregnant women	1922	1. 64.5% of pregnant women had taken ≥3 IPTp-SP doses<br>2. 15.5% had received 2 IPTp-SP doses<br>3. 6% had taken 1 IPTp-SP dose			Taking ≥3 IPTp-SP doses was associated with an average birth weight increase of more than 0.165 kg.		
[43]<br>Malawi	To measure the extent of SP/IPT use during pregnancy in Blantyre District.	Cross-sectional design	Pregnant women	391	75.7% received at least once.<br>41.2% received SP/IPT twice or more	Multigravid women were less likely to complete the recommended dose				

[79] Malawi	To evaluate the impact of a 2-year programme for community-based delivery of sulfadoxine-pyrimethamine (SP) on intermittent preventive treatment during pregnancy coverage, antenatal clinic attendance and pregnancy outcome.	RCT	Women in catchment villages around Chikwawa and Ngabu Government Hospitals,	194		Education and counselling on malaria control and the importance of attending ANC.<br><br>Community distribution of sulfadoxine-pyrimethamine for intermittent preventive treatment of malaria.					
[113] Democratic Republic of Congo	To assess the effect of intermittent preventive treatment with sulfadoxine–pyrimethamine<br><br>(IPTp-SP) on birth weight in sites with varying	Cross-sectional survey	Pregnant women	1393				IPTp-SP was effective in reducing the risk of low birth weight			

	degrees of drug resistance.									
[72] Burkina Faso	To assess the efficacy at individual level of intermittent preventive treatment with sulfadoxine–pyrimethamine (IPTp-SP) in primi- and secundigravidae in rural Burkina Faso.	RCT	pregnant women	1883				The risk of malaria infection was significantly reduced by IPTp with SP in primi- and Secundigravidae after 2 or more doses. Anaemia at delivery and low birth weight was also significantly reduced		
[96] Tanzania	To assess the effectiveness of IPTp in two areas with different malaria transmission	Longitudinal/cohort	pregnant women in two health facilities in areas with high and low malaria	350				IPTp-SP reduced the risk of placental malaria.		

	intensities.		transmission intensities							
[65] Burkina Faso, Ghana, Mali, Malawi, Kenya, Nigeria, Sierra Leone, and Uganda	To measure the prevalence of IPTp uptake in selected malaria-endemic countries in sub-Saharan Africa, and to investigate the patterns of IPTp uptake among different educational and wealth categories adjusted for relevant sociodemographic factors.	Cross-sectional study	Women aged between 15 and 49 years	18,603	The overall rate of taking three doses of IPTp-SP in the latest pregnancy was 29.5%.	Women with higher educational level were less likely to take IPTp-SP<br>Women in the richest households were more likely to take IPTp-SP compared to those in the poorest, poorer, middle, and richer households	-	-	-	-
[35] Sierra Leone	To assess the prevalence and predictors of IPTp-SP uptake in pregnancy in Sierra Leone	Cross-sectional study	Women aged between 15–49 years	8526	At least 3 doses = 93.24%	Adequate antenatal care visit increased the odds of taking IPTp-SP	Women with higher education, higher parity > 4 were less likely to take IPTp-SP			

[44]<br><br>Ghana	To determine benefits associated with intermittent preventive treatment (IPTp), antenatal care, and/or bed net use during pregnancy	Longitudinal/cohort	Pregnant women in rural north-eastern Ghana	2,232	> 82% received at least one dose of IPTp-SP during their gestation			In newborns of primigravida, greater birth weight, newborns, improved haemoglobin levels, and less anaemia were recorded.		
[114]<br><br>Ghana	To evaluate the effectiveness of IPTp-SP among pregnant women attending the antenatal clinic at Korle-Bu Teaching Hospital in Accra	Cross-sectional study	Pregnant women	363				IPTp-SP was effective evidenced by 15.3% of IPTp users had malaria compared with 44.7% of non-IPTp users.<br><br>Also, 58.4% of non-IPTp users were anaemic		

								compared with 22.8% of IPTp users		
[99] Nigeria	determine the prevalence of asymptomatic malaria parasitaemia in pregnant women on intermittent preventive treatment (IPT) with sulfadoxine pyrimethamine (SP) compared with a control group	Longitudinal/ study	Pregnant women at between 14–24 weeks gestation	363				IPT with SP during pregnancy did not significantly reduce the prevalence of the malaria parasitaemia among the pregnant women		
[110] Côte D'Ivoire	To investigate the effectiveness of IPT-SP against malaria in pregnant women within Cote d'Ivoire (San-Pedro) following 10 years of inception	cross-sectional study	Pregnant women	197				Women who took three doses of IPT-SP were less infected at the cord, placental and venous level.		

	via molecular methods.									
[111] Nigeria	To assess the effectiveness of IPTp-SP in preventing maternal malaria and malaria-associated anaemia and other contributory factors among pregnant women attending antenatal clinic.	Cross-sectional study	Pregnant women	109				IPTp-SP users were associated with negative malaria test than non-users. Haemoglobin level was higher in IPTp-SP users than non-users		
[36] Zambia	To determine the prevalence and predictors of the uptake of four or more doses of sulfadoxine	A cross-sectional survey	women of reproductive age (15–45 years) who gave birth within the 5 years before the	3686	7.5% (uptake of 4 or more doses)	The uptake of adequate doses of SP was highest for women in the low wealth percentile (11.4 %). Place of residence,				

	py-rimethamine (IPTp-SP 4+) in Zambia		survey			women from rural areas had higher (7.6%) uptake of adequate doses compared with women from urban areas (7.2 %)<br><br>Knowledge about malaria prevention<br><br>Women who had knowledge about malaria prevention had significant lower odds of taking an adequate IPTp-SP				
[30]<br><br>Ghana	to evaluate the impact of additional SP doses<br><br>on placental malaria, other malaria indicators, birth outcomes and<br><br>safety, among pregnant	Longitudinal/Cohort	pregnant women enrolled<br><br>at antenatal care (ANC) clinics	1926	5.3%≤1 dose,<br><br>19.2%≤2 doses<br><br>33.2% ≤3 doses<br><br>42.3%≤4 doses			The prevalence of active placental infection was highest<br><br>in women who received<br><br>≤<br><br>1dose (6.9%),		Miscarriages 0.5% (10/19260)<br><br>Still birth 0.9 (17/1926)<br><br>Low birth weight 7.5% (140/1856)

	Ghanaian women and their new borns							followed by those who received 2 doses (4.1%) but the same, 2.2% in each case, among those who received 3 or ≥4 doses of IPTp-SP		
[119] Uganda	To assess whether traditional birth attendants, drug-shop vendors, community reproductive-health workers, or adolescent peer mobilizers could administer intermittent preventive treatment (IPTp) for	RCT	Pregnant women	2785	1905 (92.4%) of 2062 received first dose of SP in the second trimester compared with 523 (76.1%) of 687 receiving treatment in health units.			Decreased reported malaria episodes at both delivery systems. The prevalence of anaemia by number of IPTp-SP doses received was 40.4% (40/90), 38.8%	Death 4/1130 (0.4%) = new delivery system.	Abortion<br>Still birth.<br>Low birth weight<br>Death

	malaria with sulfa-doxine-py-rimetham-ine to preg-nant<br><br>women							(130/335) 35.5% (206/581)<br><br>and 30.0% (222/741) in women who re-ceived ≤1, 2, 3<br><br>and ≥4 doses of IPTp-SP respec-tively.<br><br>Lower propor-tion of anaemia<br><br>no preva-lence of jaundice and high-est hae-moglobin concentra-tion in ba-bies who received ≥4 doses of SP dur-ing preg-nancy		

[124]<br><br>Mozambique	To evaluate the safety and efficacy of two intermittent<br><br>doses of SP in women of all parities and regardless of HIV status,<br><br>who had been given a long-lasting ITN (LLITN) through the<br><br>antenatal clinic (ANC)	RCT	Pregnant women	1030					Vomiting, mild skin reaction	Death<br><br>Spontaneous abortion, still birth, low birth weight.
[47]<br><br>Malawi	To evaluate the impact of IPTp-SP on decreasing malaria parasitaemia	Longitudinal/Cohort	Pregnant women,	8,131	one dose of SP<br><br>IPTp increased from 77.0% in<br><br>1997 to 95.3% in 2004.<br><br>second dose,<br><br>coverage increased from 27.0% in 1997			The number of SP IPTp doses was<br><br>associated with protection against placental parasitaemia, maternal		

					to 77.8% in 2004.<br><br>Coverage decreased in 2005 and 2006			anaemia and LBW, and with increases in mean maternal<br><br>haemoglobin levels and birth weights.			
[63]<br><br>Benin	To assess the benefit of IPTp-SP with respect to those of chloroquine	Cross-sectional survey	12 weeks postnatal women seeking care.	255	Full dose (14.5%).	Four ANC visits or more and high stock levels.	Receiving first dose in the third trimester.	Significantly decreased risk of delivering an infant with low birth weight<br><br>Decreased risk of placental infection			
[129]<br><br>Nigeria	To assess the use of IPTp among pregnant women attending primary health centres in the rural area and determine	Cross-sectional survey	Pregnant women	209	At least 1 dose = 27.3%						

	factors that influence the uptake									
[45]<br><br>Gabon	To determine the level of ANC attendance and its relationship with IPTp-SP and bed net coverage in Gabonese pregnant women.	Cross-sectional survey	Pregnant women	1030	84.1% received IPTp-SP<br><br>57.4% received at least 2 doses	Increased ANC attendance				
[97]<br><br>Nigeria	To evaluate the effectiveness of IPT-SP in the prevention of maternal and placental malaria in parturient mothers in Ibadan, Nigeria, where the risk of malaria is present all year round.	Longitudinal/Cohort	Pregnant women	983				IPT-SP was effective in preventing maternal and placental malaria.<br><br>It was also effective in reducing low birth weight and anaemia in the mothers		
[121]	To evaluate the effect of	RCT	Pregnant women	302				IPTp was associated		

Mozambique	IPTp on the humoral immune responses of mothers at delivery							with a reduction in antibodies in HIV-infected women.		
[93]<br><br>Tanzania	To assess the KAP on IPTp-SP	Qualitative design	Key informants, nurses, and pregnant women	77	Low		Perception that IPTp-SP has adverse effects on<br><br>Pregnant women and being uniformed or misinformed about the standard of dosage		Burning of the skin<br><br>General body weakness	
[53]<br><br>The Gambia	To examine the differentials in prevalence and correlates on the uptake of tetanus toxoid and<br><br>intermittent preventive treatment of malaria among pregnant women in the Gambia	Cross-sectional survey	Pregnant women	6143	34.3% (at least three doses)	Age (20–24 and 25–29), Parity (0-3),<br><br>use of radio, use of newspaper, ANC visits, rural resident, Married women, No education, Poorest households wealth index				

						.				
[103]<br><br>Mali	To compare the efficacy and safety of 3-dose versus 2-dose IPTp-SP for the prevention of placental malaria and associated low birth weight (LBW).	RCT	Pregnant women	814				2 -3 doses<br><br>of IPTp-SP were associated with reduced placental malaria, LBW, and preterm births		
[68]<br><br>Mali and Burkina Faso, Zambia, Malawi, Uganda and Kenya	To investigate the efficacy of IPTp-SP at clearing existing peripheral infections in asymptomatic pregnant women receiving their first dose of IPTp-SP	Longitudinal<br><br>Study/Cohort	Pregnant women	1222		High-resistance areas,<br><br>Use of ITNs the night prior to enrolment		IPTp-SP was associated with a 22% reduction in the risk of LBW.		

[105] Burkina Faso, The Gambia, and Benin	To establish whether adding community-scheduled malaria screening and treatment (CSST) by CHWs to standard IPTp-SP would further reduce placental malaria compared to IPTp-SP alone	RCT	Pregnant women	2,500				Increasing number of IPTp-SP doses was associated with a significantly lower risk of placenta malaria, anaemia at delivery, and low birth weight.		
[109] Tanzania	To investigate the protective efficacy of IPTp-DHP vs. IPTp-SP against malaria in pregnancy and adverse birth outcomes.	RCT	Malaria-free pregnant women attending their first ANC.	956				The prevalence of maternal malaria at delivery was significantly lower in IPTp-DHP than IPTp-SP group. The incidence per		

								person-years at risk for symptomatic-malaria and parasitaemia during pregnancy were significantly lower in the IPTp-DHP group than in the IPTp-SP group. The prevalence of LBW was significantly lower in IPTp-DHP compared with IPTp-SP.			
[84] Burkina Faso	To evaluate factors associated with	RCT	Women in their first or second pregnancy	2,240	Complete uptake of IPTp-SP was 71.8%.	- community sensitization, -Age (> 19)					

	antenatal clinic (ANC) attendance and uptake of IPTp-SP					-Secundigravida<br><br>-Any school<br><br>-SES (least poor)<br><br>- Season of delivery (low transmission season)<br><br>-Distance from nearest health centre (0–5 km				
[101]<br><br>Côte d'Ivoire	To assess the incidence of placental malaria, low birth weight, and anaemia of two IPTp-SP approaches	Longitudinal design	Pregnant women	420				There was no difference in terms of LBW, placental malaria infection, and anaemia at delivery between the two groups.<br><br>ITNs use appeared	Nausea, vomiting, tiredness, and headaches	

								to be an important variable in determining the efficacy of IPTp-SP		
[98] Mali, Zambia, Mozambique, and Sudan	To compare the pharmacokinetic parameters of sulfadoxine and pyrimethamine in pregnant women and after delivery using nonlinear mixed-effects modelling.	Longitudinal/Cohort	Pregnant women	98				Plasma clearance increased 3-fold for sulfadoxine		
[89] Nigeria	To identify opportunities to improve uptake of IPTp.	Cross-sectional study	Pregnant women	360			-low levels of awareness on IPTp services	-		

							Low knowledge of clients on IPTp services<br><br>Some facilities are far away from the rural communities.<br><br>Very long waiting.<br><br>Frequent stock-outs in facilities.			
[40]<br><br>Tanzania	To assesses the extent and predictors of uptake of optimal doses of IPTp-SP in six districts of Tanzania	Cross-sectional study	singleton pregnant women	431	-			The uptake of three or more doses of IPTp-SP was a significant predictor of negative malaria in pregnancy using RDT.		

[78]<br>Nigeria	To assess the prevalence and determinants of IPTp utilization by pregnant women in a rural town in Western Nigeria.	Cross-sectional study	pregnant women	255	107/255 (40.4%) reported taking medications [SP] during the current pregnancy.	A determinant for utilization of IPTp was the knowledge of prophylaxis for malaria prevention.				
[82]<br>Benin	To assess the ITP-SP coverage rate in two regions in the south and north of the country from its implementation until 2009.	Longitudinal study/Cohort	Pregnant women	2420	The rate of IPT-SP coverage increased from 3.7% in 2005 to 87.8% in 2009 for women who had received at least one dose.	- Urban dwellers had better chances of receiving IPTp.<br>- women who had undergone at least four antenatal had better chances of receiving IPTp	Stock shortage of the drug in certain maternity hospitals.<br>Late consultations for certain pregnant women.	-	-	-
[88]<br>Tanzania	To assess the influence of real and perceived costs of ANC seeking on pregnant women's access to Intermittent Preventive	cross-sectional survey	Pregnant women	823	-	-	Long distance to the antenatal services.<br>Elements of user-fees charged by healthcare workers.	-	-	-

	Treatment in Pregnancy (IPTp) against malaria in two rural districts in Tanzania						Some health workers asked for bribes from the clients.			
[90] Mozambique	A pilot MiP programme in Mozambique was designed to determine requirements for scale-up.	Longitudinal/Cohort	Pregnant women	7911	-	-	Frequent absences and staffing changes mandated on-the job	-	-	-
[104] Ghana	To determine whether IST using SP or amodiaquine+artesunate (AQ+AS) is as effective in preventing maternal anaemia and low birth weight as SP-IPTp.	RCT	Pregnant women	1111	-	-	-	Preventing low birth weight<br>Preventing anaemia<br>Preventing placental malaria	-	-

[50]<br><br>Uganda	The study sought to describe the<br><br>use of SP during pregnancy in one health district in Uganda and to<br><br>identify determinants of use of IPTp.	Cross-sectional survey	Women	500	2 or more doses<br><br>of SP was 44.8%,<br><br>31.3% of women<br><br>received a full 2-dose course of IPTp-SP	Women living in a rural village.	Inadequate knowledge about the safety of SP use during pregnancy.<br><br>Lower<br><br>educational attainment.<br><br>Living more than<br><br>30 minutes walking distance to the ANC clinic<br><br>Inadequate knowledge of malaria			
[123]<br><br>Gambia, Mali, Burkina Faso, and Ghana	To investigate whether ISTp-AL is non-inferior to IPTp-SP in preventing malaria	RCT	Primps Pregnant women	2678					Miscarriage<br><br>Perinatal death	Congenital abnormality<br><br>Small for gestational age

	infection of the placenta as well as being non-inferior in the prevention of low birth weight (LBW) and anaemia.								Maternal death	Preterm birth<br><br>Stillbirths
[70]<br><br>Nigeria	To assess the effect of intermittent preventive treatment with sulfadoxine and pyrimethamine (IPT-SP) on placental parasitaemia and maternal and perinatal outcome.	RCT	Pregnant women receiving antenatal care	741		continuous training for healthcare providers to improve their knowledge, supportive supervision of healthcare providers, increased government commitment towards providing SP, education of men on the dangers of malaria in pregnancy constant SP.		There was reduction in the number of malaria cases		
[115]	To assess whether	RCT	HIV-uninfected	1617				IPTp-SP effective in		

Kenya and Uganda	piperaquine shows greater antimalarial benefits for birth outcomes than does sulfadoxine-pyrimethamine.		pregnant women					reducing placental malaria infection,		
[32] Ghana	To evaluate the effect of SP use in pregnancy on malaria–associated maternal morbidity and neonatal birthweight	Mixed method	pregnant women attending antenatal clinic	487	24%, 15% and 9% respectively took 1, 2 and 3 doses of SP.		lack of attendance to ANC	reduction of maternal anaemia and parasitaemia	general malaise, body weakness, nausea, vomiting, palpitations, and itching.	
[64] Cameroon	To determine IPTp-SP adherence and coverage, and the impact on	Cross-sectional survey	Pregnant women attending the health facilities for antenatal care (ANC)	888	17.5%			Reduced maternal anaemia, reduced LBW, reduced infection		

	maternal infection and birth outcomes in the context of wide-spread SP resistance		visits and delivery.					among newborns		
[58] Tanzania	To evaluate the effectiveness of IPTp-SP given as DOT and monitored the treatment outcomes	Longitudinal/Cohort	Pregnant women	500	61.4% (more than or equal to three doses), 38.6% (less than three doses).			reduced risk of having placental malaria at delivery		
[66] Nigeria	to identify the barriers to and determinants of the use of SP-IPTp among pregnant women attending ANC in	Cross-sectional study	pregnant women	400	41 %		lack of autonomy or freedom to receive IPTp-SP during ANC without consulting a household member, stock-outs of free SP, poor			

	PHC facilities						implementation of directly observed treatment in the ANC clinic, late ANC attendance and poor knowledge of the gestation			
[67] Tanzania	to evaluate the protective efficacy and safety of three different antimalarial regimens for intermittent preventive treatment in infants	RCT	infants aged 8-16 weeks	2419	>97%			did not show any significant reduction in episodes of clinical malaria		
[41] Cameroun	To assess malaria risk factors in women on intermittent preventive treatment with SP (IPTp	Cross sectional Survey	Pregnant women	201	38%, 20% and 6% participants took one, 2 and 3 doses respectively.	ANC attendance by the mother<br>Higher Educational level of mothers		Reduction in the prevalence of anaemia with the use of SP in		

	-SP) at delivery<br><br>and their effects on pregnancy outcome							primiparous women		
[34]<br><br>Uganda	To assess the effectiveness of IPTp with SP in reducing the risk of malaria and improving birth outcomes	Cross-sectional survey	HIV- Uninfected pregnant women	565	35.8% =1 dose of SP<br><br>56.6% = 2 doses of SP<br><br>2.0%=3 doses of SP	Higher wealth index, secondary education or higher.		Taking 2 doses or more of SP lead to lower odds of placental malaria, maternal, low birth weight, maternal parasitaemia, maternal anaemia		
[108]<br><br>Benin<br><br>Gabon<br><br>Mozambique<br><br>Tanzania	To evaluate the safety and efficacy of SP and Mefloquine in HIV negative women	RCT	Pregnant women attending ANC for the first time	4749				Women in the SP group had increased risk of parasitaemia compared with those in the MQ group. Again, SP recipients had more		

								clinical malaria.		
[42]<br><br>Kenya	To evaluate if IPTp coverage increased and if the training in Asembo led to better coverage<br><br>than in Gem, and to identify barriers to the effective implementation of IPTp	cross-sectional survey	Women who have recently delivered	724 women	One dose of SP 41%, two or more doses 21%		More likely to receive less or equal to 1 dose-Being single, a history of child death,<br><br>More likely to receive less or equal to 2 doses-single women, women with less than 8 years of educational, history of child death.			
[92]<br><br>Benin	To investigate the impact of the timing of intermittent preventive treatment with Sulfadoxine-Pyrimethamine (IPTp-SP) on birth weight outcomes and placental infection risk in pregnant	RCT	Pregnant women who delivered live singletons	1439			Limited Healthcare Infrastructure, Geographic Challenges, Socioeconomic factors (financial constraints), stockouts of SP, Health System Challenges (long waiting time, inadequate counselling, or support for pregnant women),		low birth weight infants,<br><br>Increased risk of placental infection, Gestational malaria	Low Birth Weight, Increased Risk of Malaria Transmission, increased risk of developmental delays, chronic health conditions, and impaired immune function

	women in Benin.							Cultural Beliefs and Practices			
[39] Africa (Mali, Mozambique, Sudan, and Zambia).	To assess the pharmacokinetics of sulfadoxine-pyrimethamine (SP) during pregnancy and after the postpartum period to inform rational dosing of SP for intermittent preventive treatment in pregnancy.	RCT	Pregnant women receiving prenatal care	98	Pregnant women in the four African countries (a total of 98 pregnant women across four African countries: 18 in Mali, 31 in Mozambique, 24 in Sudan, and 25 in Zambia).	Pregnant women receiving prenatal care in health facilities.	Limited Availability of SP tablets, Complex Dosage Regimen of SP tablets, history of allergy to SP or other sulpha drugs, recent treatment with SP or other antimalarials, significant anaemia, and laboratory evidence of renal or hepatic impairment,		Headache, backache, toothache, gastrointestinal upset, urinary symptoms, and genital infections.	

[130]<br><br>SSA<br><br>(DR Congo, Madagascar, Mozambique, and Nigeria)	To assess the effect of community delivery of IPTp (C-IPTp) on antenatal care and IPTp coverage	Quasi-experimental<br><br>Design	Women of reproductive age (13–50 years old.	18 215	DR Congo: IPTp3+ coverage increased from 22·5% at baseline to 65·2% at endline<br><br>Madagascar:<br><br>IPTp3+ increased from 17·7% to 74·9%<br><br>Nigeria: IPTp3+ increased from 12·7% to 62·7%<br><br>Mozambique: IPTp3+ coverage increased from 51·8% to 58·6%					
[131]<br><br>Malawi	To assess	RCT	HIV-negative women with a	1561		Areas of low resistance	-	-	Vomiting	• Foetal loss

	whether IPTp with dihydroartemisinin–piperaquine, alone or combined with azithromycin, can reduce adverse pregnancy outcomes compared with IPTp with sulfadoxine–pyrimethamine		viable singleton pregnancy							• Low birth weight<br>• Neonatal death<br>• Small for gestational age
